# Senescence-induced cellular reprogramming drives cnidarian whole-body regeneration

**DOI:** 10.1016/j.celrep.2023.112687

**Published:** 2023-06-30

**Authors:** Miguel Salinas-Saavedra, Febrimarsa, Gabriel Krasovec, Helen R Horkan, Andreas D Baxevanis, Uri Frank

**Affiliations:** 1 Centre for Chromosome Biology, School of Biological and Chemical Sciences, National University of Ireland, Galway; 2 Computational and Statistical Genomics Branch, Division of Intramural Research, National Human Genome Research Institute, National Institutes of Health, Bethesda, MD 20892 USA

## Abstract

Cell fate stability is essential to maintaining ‘law and order’ in complex animals. However, high stability comes at the cost of reduced plasticity and, by extension, poor regenerative ability. This evolutionary trade-off has resulted in most modern animals being rather simple and regenerative or complex and non-regenerative. The mechanisms mediating cellular plasticity and allowing for regeneration remain unknown. We show that signals emitted by senescent cells can destabilize the differentiated state of neighboring somatic cells, reprogramming them into stem cells that are capable of driving whole-body regeneration in the cnidarian *Hydractinia symbiolongicarpus*. Pharmacological or genetic inhibition of senescence prevents reprogramming and regeneration. Conversely, induction of transient ectopic senescence in a regenerative context results in supernumerary stem cells and faster regeneration. We propose that senescence signaling is an ancient mechanism mediating cellular plasticity. Understanding the senescence environment that promotes cellular reprogramming could provide a new avenue to enhance regeneration.

## Introduction

Regenerative abilities of animals are irregularly scattered among taxa but are largely reverse correlated with their structural complexity. With some exceptions, morphologically simple animals such as sponges, cnidarians, and planarians can regenerate whole-bodies from tissue fragments while complex animals such as vertebrates have more limited capabilities to restore lost body parts.^1^ Regeneration requires a certain level of growth plasticity that can be realized by a resident pool of stem cells or by mechanisms that induce reprogramming of differentiated cells to provide proliferative progenitors.^2^ However, the high plasticity that allows for whole-body regeneration may also compromise the integrity of complex structures and increase malignancy risk.^3,4^ Therefore, high plasticity and structural/morphological complexity rarely co-occur in one species.

A major question arising from this line of arguments concerns the nature of the mechanisms that mediate cellular plasticity in regenerative animals. If regeneration was a primitive trait in metazoans,^1^ these mechanisms have been lost or became ineffective in non-regenerative taxa. Therefore, studying animals with high regenerative abilities could provide insight into the lack of regeneration in other animals. In the long term, this information might be harnessed to induce plasticity in complex, non-regenerative animals, enhancing their regenerative abilities under controlled conditions.

Studying regeneration in a cnidarian, we show that senescence signaling can drive somatic cell reprogramming in a regenerative context. Our results, combined with data from regeneration studies in other animals, suggest that senescence is an ancient mechanism in metazoans, used to convey a stress signal to surrounding tissues, thereby driving a regenerative response. We propose that the ability to respond to a senescence signal is one of the factors underlying differential regenerative abilities in modern metazoans.

## Results

### Stem cell-less tissues can regenerate whole animals that include stem cells

The cnidarian *Hydractinia symbiolongicarpus*—a relative of jellyfishes and corals—is a highly regenerative animal that is able to regrow a lost head within 3 days post amputation (dpa).^5,6^ *Hydractinia* head regeneration is driven by a population of adult, pluripotent migratory stem cells, known as i-cells,^7^ that are normally restricted to the lower body column of the animal and can differentiate into both somatic cells and gametes.^8^ These i-cells, which can easily be visualized via *Piwi1* expression, migrate to the injury site post-amputation to restore the head.

**Figure 1:**
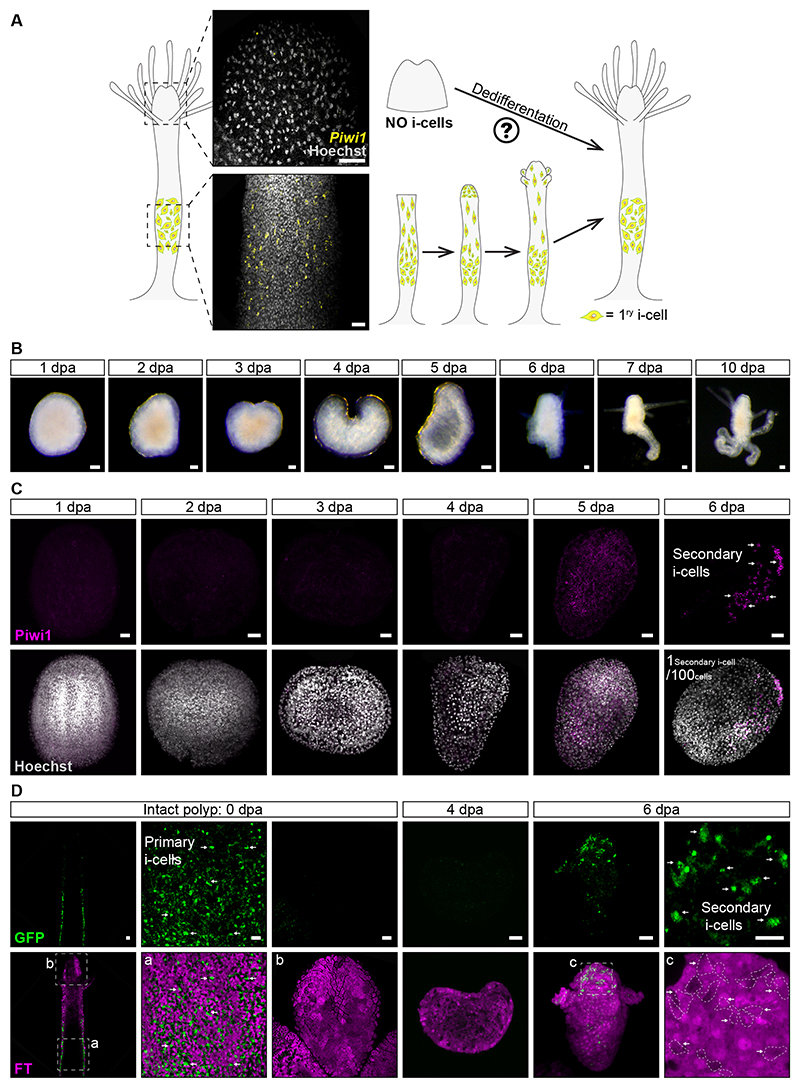
Secondary i-cells emerge in amputated hypostomes 6 dpa. **(A)** *Piwi1* mRNA SABER-FISH of an intact *Hydractinia* polyp. Primary i-cells are restricted to the lower part of the body but absent from the hypostome. **(B)** Amputated hypostomes can regenerate a fully functional animal within 6-10 dpa. **(C)** Piwi1 antibodies detect secondary i-cells in hypostomes, 6 dpa (arrows). On average, we observed 1 secondary i-cell per 100 cells. **(D)** *In vivo* imaging of primary and secondary i-cells (arrows) in a *Piwi1*::FastFT reporter animal. In the intact polyp, primary i-cells (GFP^+^) do not overlap with the FT red fluorescence and are absent from the hypostome. However, in amputated hypostomes, secondary i-cells emerging at 6 dpa (depicted with arrows and dashed lines) overlap with the FT red fluorescence (c) observed at 4 dpa. Tissues do not express GFP at 4 dpa. This shows the reprogramming of somatic cells to secondary i-cells. Scale bars: 20 *μ*m (See also Figures S1 and S2).

However, the heads of uninjured animals are devoid of i-cells (Figure 1A),^5^ so the head alone would not be expected to be able to regenerate a new body due to its lack of i-cells. Surprisingly, we have discovered that the amputated oral tips of heads (known as hypostomes) can indeed regenerate into a fully functional animal (Figure 1B) that contains i-cells despite having no i-cells immediately post-amputation (Figure 1C, S1A). We termed the i-cells that appeared *de novo* in regenerating hypostomes ‘secondary i-cells’, as opposed to primary i-cells that are generated during embryogenesis.

To visualize the process of secondary i-cell appearance *in vivo*, we generated a *Piwi1* transgenic reporter animal that expressed a Fast-FT mCherry timer protein^9^ and membrane GFP under the control of the *Piwi1* genomic control elements.^5^ Fast-FT exhibits time dependent blue-to-red chromophore maturation. The immature Fast-FP mCherry protein could not be observed under our microscopes due to the lack of a commercial filter but i-cells expressing a bright green membrane GFP could be viewed in the live translucent animal. Mature red FastFT mCherry protein was readily visible in differentiated cells (Figure 1D, S2). The reporter transgene is silenced during differentiation, resulting in the early progeny of i-cells becoming red due to maturation of the Fast-FT mCherry protein and dim green due to the GFP’s half-life (Figure 1D, S2B-C). Hypostomes were then isolated from transgenic *Piwi1* reporter animals and examined under a fluorescence microscope to exclude the presence of any bright green i-cells; these hypostomes were then incubated in seawater (Figure 1D). Consistent with the experiments described above, GFP^+^ i-cells reappeared, *de novo*, at 6 dpa in an otherwise mature red mCherry^+^ background (Figure 1D, S2D). Hence, we concluded that, following amputation, a yet unknown mechanism induces the reprogramming of differentiated somatic cells back to *Piwi1*^+^ i-cells that then drive whole body regeneration.

**Figure 2:**
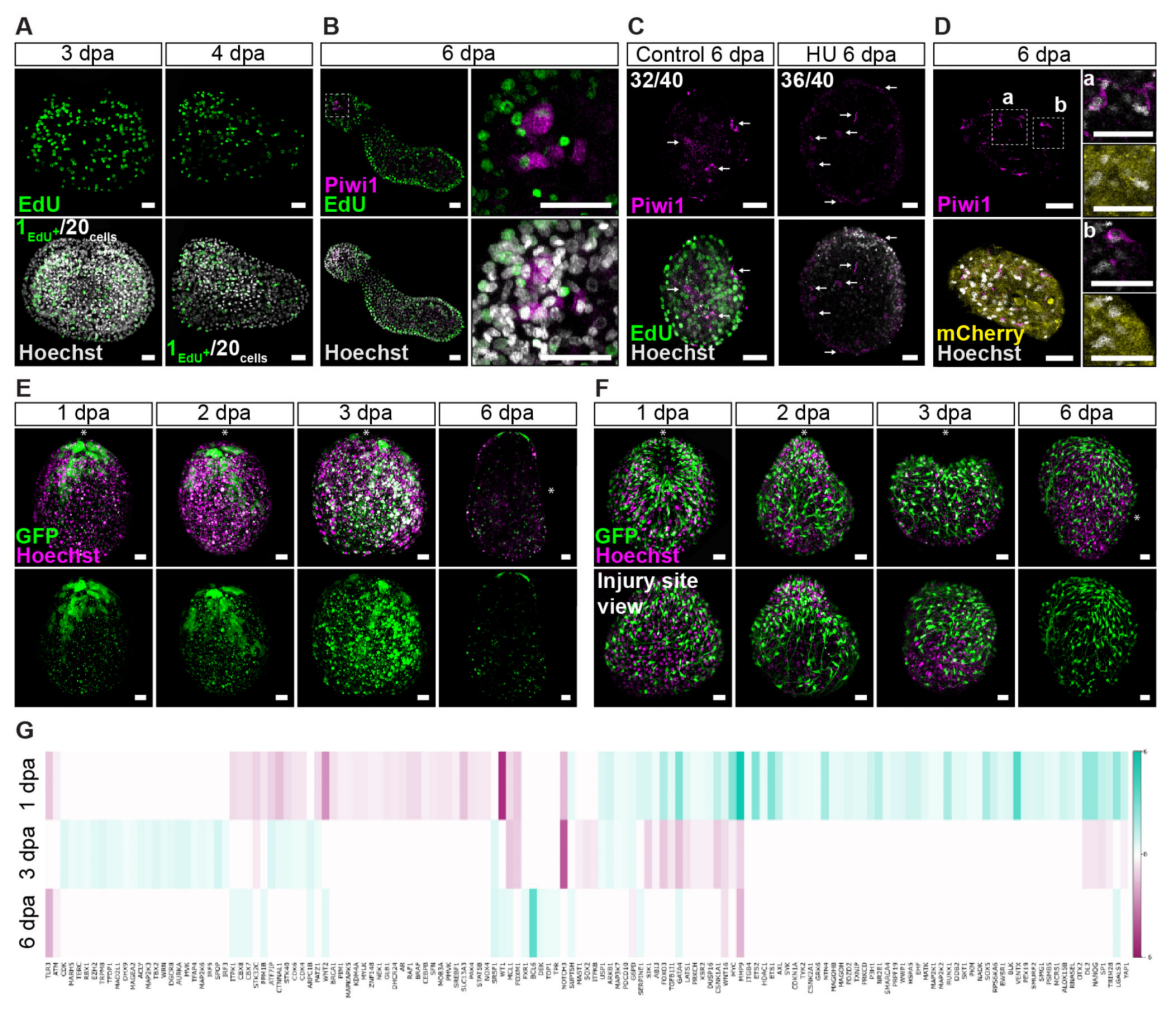
Cellular and molecular events accompanying whole-body regeneration from amputated hypostomes. **(A)** Cell cycle re-entry (EdU incorporation) takes place between 3 and 4 dpa. On average, we observed 1 EdU^+^ cell per 20 cells. **(B)** Piwi1^+^ cells have residual EdU signals at 6 dpa after being incubated in EdU for 48 hrs between 3 and 4 dpa. **(C)** only few Piwi1^+^ cells appear in hydroxyurea-treated hypostomes but do not cycle. **(D)** Piwi1^+^ cells are also mCherry^+^, indicating the reprogramming of adult somatic cells. **(E)** *In vivo* imaging of amputated hypostomes from a *Wnt3*::GFP reporter animal 1, 2, 3, and 6 dpa. Lower panels show the GFP channel only. Asterisks indicate the oral pole pre-amputation. **(F)** *In vivo* imaging of amputated hypostomes from an *RFamide*::GFP reporter animal. Lower panels show the site of amputation. Asterisks indicate the oral pole pre-amputation. **(G)** Heatmap showing senescence-associated genes are highly differentially expressed at 1 dpa and less so at 3- and 6 dpa. Scale bars: 20 *μ*m (See also Figures S1 and S3).

Intact *Hydractinia* hypostomes lack i-cells and cycling cells.^5^ Based on EdU incorporation, we established that a wave of DNA synthesis occurs between 3-4 dpa (Figure 2A, S1B). Secondary Piwi1^+^ i-cells appeared in the hypostome tissue around 6 dpa (Figure 1). These cells had residual EdU signals, showing that they were derived from cells that were in S-phase two days earlier (Figures 2B and S1B) Treating hypostomes with hydroxyurea to arrest cells in S-phase did not completely abolish the upregulation of Piwi1 in some cells (Figure 2C). However, the level of Piwi1 expression in these cells was low as compared to i-cells assessed using immunofluorescence. Moreover, hydroxyurea-treated hypostomes did not regenerate and subsequently died, showing that proliferation occurring before secondary i-cell appearance is essential for complete reprogramming of somatic cells to fully functional i-cells.

To verify that somatic cells undergo reprogramming to give rise to secondary i-cells, we amputated hypostomes from a *β-tubulin::FastFT* transgenic reporter animal. These animals express mCherry in all differentiated cells but not in i-cells.^8^ We allowed the hypostomes to develop secondary i-cells, then fixed and stained them with antibodies raised against Piwi1 and mCherry. We found cells that were double-positive, showing that cells that had expressed mCherry in the transgenic reporter animal (i.e., differentiated cells) reprogrammed into Piwi1^+^ i-cells (Figure 2D and S3A).

The *Hydractinia* main body axis is generated and maintained by Wnt/β-catenin signaling.^10,11^ We used a *Wnt3* transgenic reporter animal that expresses GFP in the oral-most tip of the hypostome^8^ to dynamically follow the polarity of the isolated hypostomes. We found that GFP expression in the reporter animal lost its oral focus within 2-3 dpa, spreading around the tissue (Figures 2E and S3B, C). We repeated the experiment with an *Rfamide* transgenic reporter animal that expresses GFP in a stereotypic fashion in the nervous system of the head.^7^ As with the *Wnt3* reporter, within 2-3 dpa, the GFP^+^ neurons lost their typical oral orientation and became disorganized (Figure 2F and S3D, E). We concluded that axis polarity is lost in isolated hypostomes within 3-4 dpa. Following secondary i-cell appearance, the hypostomes elongated and regained polarity, as visualized by *Wnt3* and *Rfamide* expression in transgenic reporter animals (Figures 2E-F and S3C, E; 6 dpa), developing into intact yet small animals.

### Senescence precedes regenerative reprogramming

We then aimed to identify the nature of the signal that induces the appearance of secondary i-cells in isolated hypostomes that were initially devoid of i-cells. We hypothesized that this signal is present in regenerating tissues a few days before both reprogramming and the emergence of secondary i-cells. Therefore, we extracted RNA from amputated hypostomes 0, 1, 3, and 6 dpa and sequenced their transcriptomes. Using CellAgeDB ^12^ as a reference, we collected sequences of 279 cell senescence-associated proteins from UniProt. We identified *Hydractinia* homologs for 229 of these proteins using reciprocal BLAST. Of these 229 homologs, we found 142 genes were significantly differentially expressed in at least one time point when compared to the preceding time point. We plotted the results of the candidate gene search as a heatmap (Figure 2G), identifying a burst of differential expression in cell senescence-associated genes on 1 dpa, which is reduced 3 dpa and further reduced 6 dpa (File S1).

Senescence is a form of irreversible cell cycle arrest in animals, induced by stress, damage, oncogene expression, or telomere attrition.^13,14^ Senescent cells secrete a cocktail of factors, collectively known as the senescence-associated secretory phenotype (SASP);^15^ these factors induce inflammation and enhance senescence and malignancy in neighboring cells.^15,16^ Long-term accumulation of senescent cells in tissues is thought to contribute to organismal aging.^17^ Conversely, a role for senescence (and short-term senescence in particular) in cellular plasticity has been reported in mammals and other vertebrates.^16,18–20^ However, its natural context of action is not well-understood. We hypothesized that amputation injury induces senescence in some cells that then emit a signal (through SASP) that promotes reprogramming of neighboring somatic cells. This would, in turn, produce stem cells that could initiate a regenerative process.

**Figure 3:**
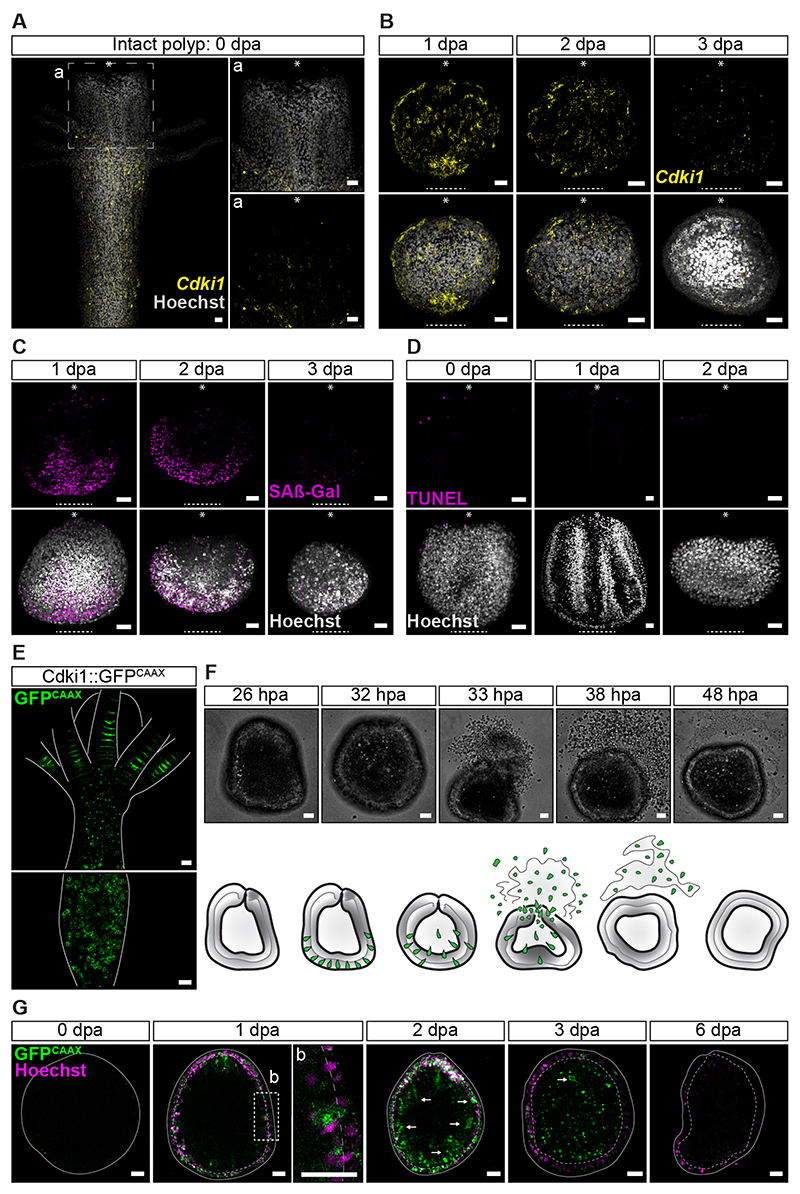
Senescence markers are transiently upregulated 1 dpa. **(A)** *Cdki1* mRNA SABER-FISH of an intact *Hydractinia* polyp. Low levels of *Cdki1* mRNA are present in the hypostome. **(B)** *Cdki1* mRNA SABER-FISH of amputated hypostomes 1, 2, and 3 dpa. *Cdki1* mRNA is upregulated at the site of injury (dashed line) at 1 dpa and dissipates 3 dpa. Asterisks indicate the oral pole pre-amputation. **(C)** SPiDER-βGal staining of amputated hypostomes 1, 2, and 3 dpa. βGal activity resembles *Cdki1* mRNA expression pattern at the site of injury (dashed line). Asterisks indicate the oral pole pre-amputation. **(D)** Absence of apoptosis (TUNEL) at the site of injury (dashed line). Asterisks indicate the oral pole pre-amputation. **(E)** *In vivo* imaging of an intact *Cdki1*::GFP^CAAX^ transgenic reporter animal. **(F)** Time-lapse imaging (extracted from Movie S1) of an amputated *Cdki1*::GFP^CAAX^ reporter hypostome at 26, 32, 33, 38, and 48 hpa. Interpretation of the images are shown as cartoon. **(G)** Time-lapse imaging of amputated hypostomes from a *Cdki1*::GFP^CAAX^ reporter animal showing the migration of senescent cells from the epidermis to the gastrodermis (arrows). No *Cdki1*::GFP^CAAX^ cells are present 6 dpa. Scale bars: 20 *μ*m (see also Figure S4, S5, and S6).

Following this line of reasoning, we looked for indicators of senescence in regenerating hypostomes. Cell cycle regulators such as p21 (encoded by *CDKN1A*) and p16 (encoded by *CDKN2A*) are known senescence markers in mammals.^21^ The *Hydractinia* genome encodes three *CDKN1A*-like genes (Figure S4 and S5) but no *CDKN2A*, the latter being vertebrate-specific. Our phylogeny could not resolve orthology between cnidarian and bilaterian CDKN1A proteins, suggesting that the *Hydractinia* genes were paralogs. Therefore, we called the *Hydractinia* three *CDKN1A*-like genes *Cyclin-dependent kinase inhibitor 1* (*Cdki1; HyS0010.253*), *Cdki2 (HyS0001.744)*, and *Cdki3 (HyS0029.124)*, respectively. Single-molecule fluorescence mRNA *in situ* hybridization showed that the three genes were expressed in some cells along the body column (Figures 3A and S5A) but only *Cdki1* was upregulated at the cut side of isolated hypostomes around 1 dpa (Figure 3B), being nearly undetectable in intact hypostomes (Figure 3A) and in the blastema of regenerating polyps (Figure S6). Expression patterns of *Cdki2* and *Cdki3* did not visibly change following injury. An additional senescence indicator is senescence-associated β-galactosidase (SAβ-Gal) activity.^21^ Using SPiDER-βGal, a cell permeable fluorescent β-galactosidase probe, we found high SAβ-Gal activity around the injury site of hypostomes, concomitantly with *Cdki1* expression (Figures 3C and S6A). The above signals reached a peak around 1 dpa and vanished 2-3 dpa, indicating a transient, short-term senescence episode at the injury site post amputation.

In the closely related cnidarian *Hydra*, it has been shown that bisection induces apoptosis in i-cells, with these dying cells emitting a Wnt3 signal that drives proliferation and head regeneration.^22^ However, in *Hydractinia* regenerating hypostomes, no evidence for apoptosis was found using TUNEL staining (Figures 3D, S4B, and S4C), suggesting that senescence-induced regeneration in the absence of stem cells is distinct from apoptosis-induced repair where i-cells are present.

**Figure 4:**
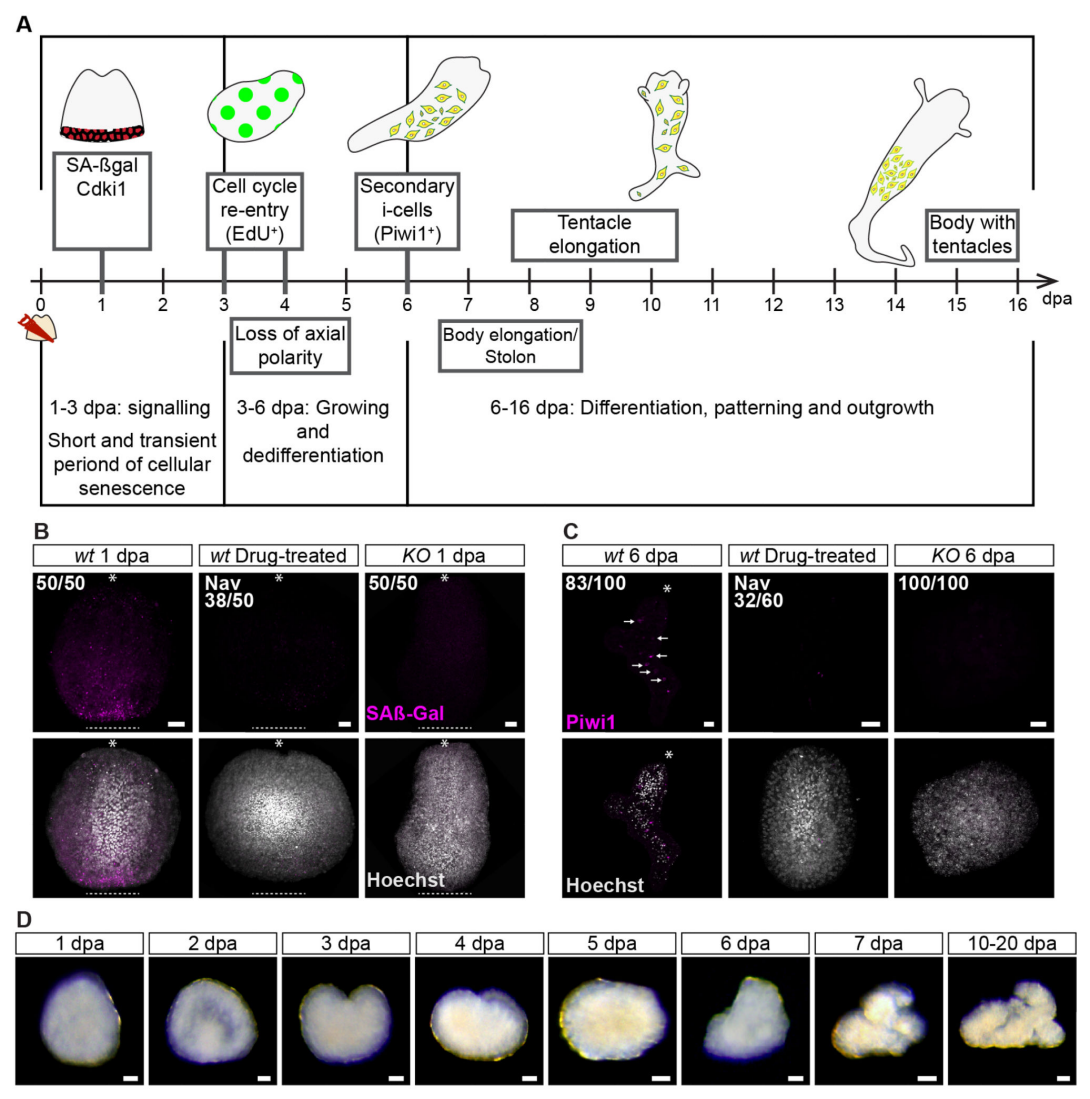
Transient senescence 1 dpa is required for somatic cell reprogramming. **(A)** Diagrammatic illustration of the major events accompanying whole-body regeneration from amputated hypostomes. **(B)** Hypostomes amputated from both Navitoclax-treated wild type and *Cdki1* KO animals do not develop cellular senescence at the site of injury 1 dpa (dashed line). Asterisks indicate the oral pole pre-amputation. **(C)** Hypostomes amputated from Navitoclax-treated and *Cdki1* KO animals do not develop Piwi1^+^ cells (arrows) at 6 dpa. Asterisks indicate the oral pole. **(D)** Amputated hypostomes from *Cdki1* KO animals do not regenerate within 20 dpa. Scale bars: 20 *μ*m (see also Figure S7 and S8).

Given that senescent cells do not spontaneously die or normally exit senescence, the disappearance of senescence markers between the second and third dpa prompted us to investigate their fates. For this, we generated a *Cdki1* transgenic reporter animal that expressed membrane GFP^CAAX^ under the *Cdki1* genomic control elements. GFP^CAAX^ fluorescence in transgenic animals faithfully recapitulated *Cdki1* mRNA expression (Figure 3E), being upregulated at the injury site 1 dpa (Figures S5B-C). We amputated hypostomes from these animals and subjected them to *in vivo* time-lapse imaging (Figures 3F and S5D; Movie 1). Strikingly, these experiments showed that epidermal senescent cells translocate to the gastrodermal tissue (Figure 3G) and are then expelled, probably through the mouth, by regenerating hypostomes between 30-40 hours post amputation (hpa; Figures 3F-G, S5C-D), consistent with the disappearance of senescence markers (Figure 3C). Therefore, whole-body regeneration from isolated hypostomes is accompanied by a short senescence period around 1 dpa, loss of tissue polarity within 3 dpa, a burst of proliferation around 3-4 dpa, and secondary i-cell appearance 6 dpa (Figure 4A). The process is completed by the re-establishment of tissue polarity, elongation, and morphogenesis.

### Senescence signaling is required and sufficient to induce reprogramming

To identify a functional link between the short period of senescence and the subsequent reprogramming of somatic cells, we used navitoclax, a senolytic drug ^23^, to inhibit senescence and study the effect of this manipulation on somatic cell reprogramming and secondary i-cell appearance. We exposed freshly amputated hypostomes to 1 µM navitoclax in seawater and followed them over 6 dpa. We found that navitoclax at this concentration inhibited senescence marker upregulation for the duration of treatment (Figures 4B and S7A). Furthermore, no secondary i-cells appeared in treated hypostomes at 6 dpa (Figure 4C). We repeated the experiments with rapamycin, an mTOR inhibitor, which yielded similar results (Figure S7B-C).

To further address the requirement of senescence to reprogramming and exclude an unspecific effect of senolytic drugs, we used CRISPR-Cas9 to mutate the *Cdki1* gene (Figure S8A). Three short guide RNAs (sgRNAs) targeting the nuclear localization signal (NLS) and PCNA-interacting domain of *Cdki1* were designed and injected with recombinant Cas9 into fertilized eggs ^24^. G0 embryos were allowed to develop into larvae, metamorphose, and grow to the young colony stage. They were then screened for mutations by genomic PCR and sequencing. Confirmed mosaic mutants were grown to sexual maturity and crossed with wild type animals. Heterozygous G1 animals were identified by PCR and sequencing, grown to sexual maturity, and interbred to give rise to G2 homozygous knockout (KO) animals (Figure S8A). These animals developed normally to the larval stage, metamorphosed, and grew to apparently normal colonies that were able to regenerate amputated heads similar to those seen in wild type animals (Figure S8B). However, EdU analysis revealed that *Cdki1* KO animals had an abnormal, broader distribution of cycling cells, consistent with the loss of a cell cycle regulator (p<0.0001, n=20; Figures S8C-D; File S2).

We amputated hypostomes from *Cdki1* KO animals and analyzed their behavior. We found that the absence of functional Cdki1 resulted in the loss of the senescence response at 1 dpa (Figure 4B) and absence of secondary i-cells at 6 dpa (Figure 4C). With the lack of i-cells, the KO hypostomes had not regenerated within 20 dpa (Figure 4D), remaining as amorphous tissue lumps that eventually died of starvation. Therefore, a short senescence signaling is essential for reprogramming.

**Figure 5:**
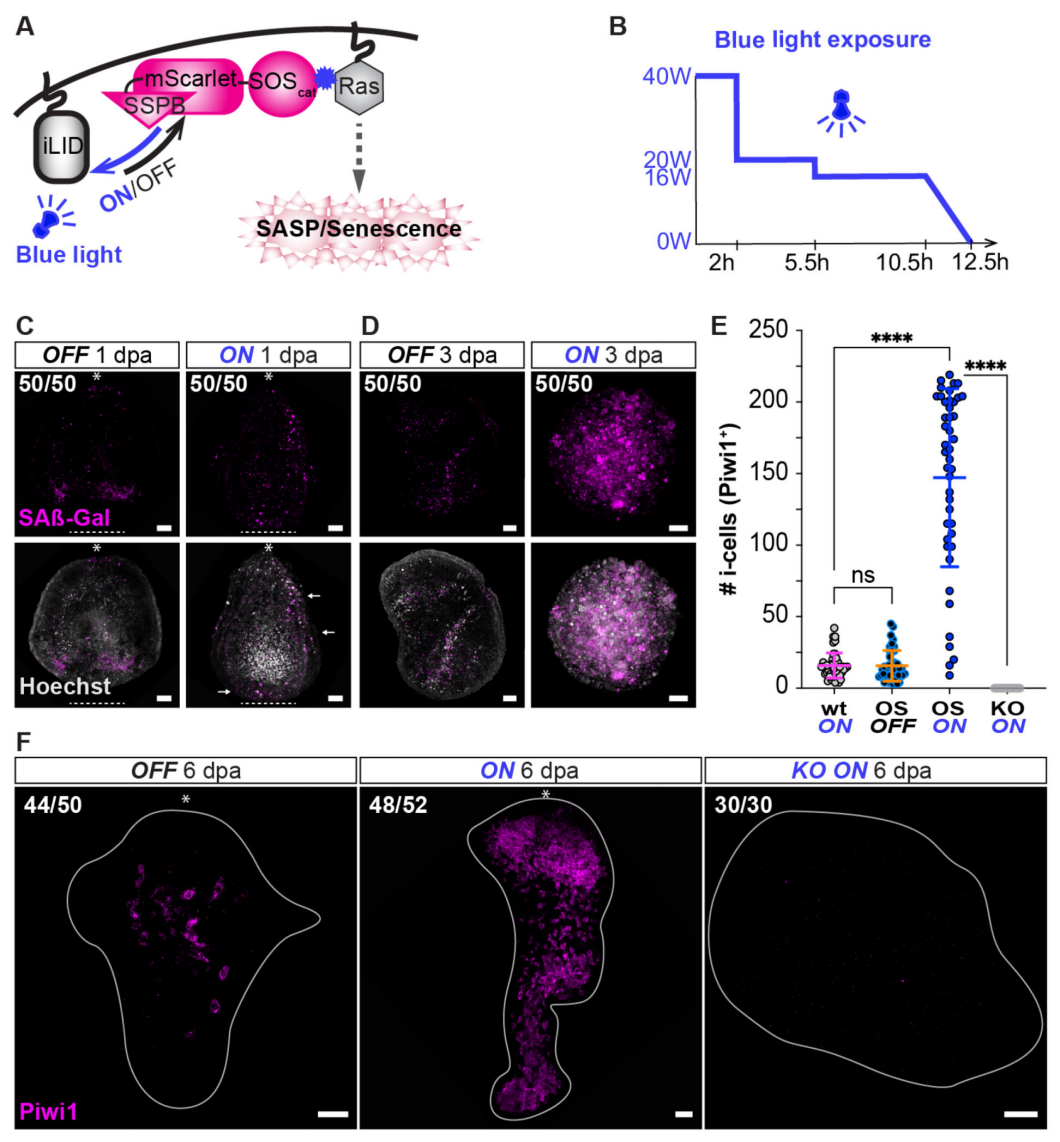
An optogenetic approach for ectopic induction of senescence during regeneration results in increased numbers of secondary i-cells. **(A)** A diagrammatic representation of the optogenetic activation of the Ras pathway when exposed to blue light. **(B)** Blue light exposure pattern during the experiments. **(C)** Ectopic senescence outside the site of injury at 1 dpa (dashed line) under blue light exposure. Asterisks indicate the oral pole pre-amputation. **(D)** Enhanced cellular senescence 3 dpa under blue light exposure. **(E)** Quantification of the number of secondary i-cells at 6 dpa (p<0.0001; wt and OS n=44; KO n=30). **(F)** Supernumerary secondary i-cells at 6 dpa under blue light exposure. Amputated hypostomes were incubated during 1 dpa under four different conditions: wild type under blue light exposure (wt ON), OptoSOS under darkness (OS OFF), OptoSOS under blue light exposure (OS ON), *Cdki1* KO OptoSOS under blue light exposure (KO ON). Scale bars: 20 *μ*m (see also Figure S9).

Finally, we tested the ability of ectopic senescence to induce reprogramming in this specific context. To induce senescence, we employed an optogenetic approach using the Opto-SOS genetic cassette.^25^ Cells carrying this construct respond to blue light by overactivation of the Ras pathway (Figure 5A), and overactivation of Ras is known to induce senescence.^26^ We generated transgenic mosaic animals that carried the Opto-SOS construct, fused to mScarlet. Hypostomes were amputated and exposed to blue light for 12 hpa (Figure 5B) and we then observed the events accompanying their regeneration. As expected, we found that exposure to blue light (which caused Ras pathway activation through the Opto-SOS construct) induced enhanced SAβ-Gal activity (Figures 5C-D), and secondary i-cells appeared 5 dpa (Figure S9A) as opposed to 6 dpa in animals kept in the dark; animals exposed to blue light grew faster than control ones (Figure S9A). At 6 dpa, the number of secondary i-cells was significantly enhanced in animals exposed to blue light comparing to those kept in the dark (p<0.0001, n=44; Figure 5E, F; Supplemental File 2. To confirm the involvement of Cdki1-mediated senescence signaling and exclude a non-specific effect of the Opto-SOS construct, we treated hypostomes with Navitoclax. This caused a reduction in the numbers of secondary i-cells (Figure S9B-C). Finally, we generated *Cdki1* KO animals that carried the Opto-SOS construct. However, after exposing them to blue light (Figure 5E, F), no i-cells were identified in their tissues. Taken together, a signal emitted by senescent cells that are transiently present following injury is essential and sufficient to induce the reprogramming of somatic cells to stemness in amputated hypostomes.

## Discussion

Animal somatic cells face contradictory requirements during the life history of individuals. On the one hand, cell fate stability is needed to maintain structural integrity and prevent malignancy while, on the other hand, stably differentiated cells prevent regeneration. Extant animals appear to deal with this problem using a spectrum of strategies. Morphologically simple animals such as cnidarians possess high cellular plasticity and regenerative powers. They can tolerate the presence of unstable cells because their structural organization facilitates the shedding of unwanted cells (Figures 3F-G). By contrast, morphologically complex bilaterians such as mammals cannot afford to harbor developmentally plastic cells due to malignancy risk and the necessity to maintain complex structures. The results presented here are consistent with the notion that senescence signaling is one of the factors that mediate cellular plasticity.^13,27^ Recently, studies using salamanders have shown a similar role for senescence in limb regeneration.^28,29^ However, the ability to respond to a senescence signal by inducing reprogramming has not been highly conserved across lineages during metazoan evolution.

A rudimentary response to senescence signals by increased plasticity is still present in modern mammals. This has been shown in mouse liver cells ^18^, skeletal muscles,^30^ and by the discovery that a senescent environment facilitates reprogramming by OSKM factors.^31^ Finally, embryonic and induced pluripotent stem cells maintain pluripotency when grown on a feeder layer consisting of senescent fibroblasts.^32^ However, except for urodele amphibians,^19^ tetrapod vertebrates have poor regenerative ability and do not respond to a senescence signal as effectively as *Hydractinia*.

We suggest that senescence is an ancient mechanism, instructing cells adjacent to an injury site to prepare for a regenerative event. We also speculate that other consequences of senescence that have been observed in mammals, such as long-term retention and accumulation of senescent cells, aging, chronic inflammation, and cancer, are side effects that evolved later in the evolution of these lineages, perhaps as consequence of the increase in cell fate stability and morphological complexity. Understanding the senescent environment and its role in cellular plasticity could pave the way for new treatments to enhance regeneration in poorly regenerating mammals.

### Limitations of the study

Our study provides strong evidence for a role for senescence signaling in cellular reprograming in cnidarians. While similar phenomena have been observed by others in other animals, the degree to which components of the senescence signaling pathway are evolutionarily conserved across phyla is at present unclear. Markers for cellular senescence are not universal and no definitive marker for senescence has been identified. Moreover, the existence of different senescence ‘types’ has been proposed.^33^ Finally, we have not been able to show that secondary i-cells are pluripotent as are primary i-cells.

## STAR Methods

### Resource Availability

#### Lead Contact

Further information and requests for resources and reagents should be directed to and will be fulfilled by the Lead Contact, Uri Frank (uri.frank@universityofgalway.ie)

#### Materials Availability

This study did not generate new unique reagents.

#### Data and Code Availability

- The *Hydractinia symbiolongicarpus* genome is available in the *Hydractinia* Genome Project Portal through the NIH National Human Genome Research Institute of the National Institutes of Health (https://research.nhgri.nih.gov/hydractinia/). Corresponding data are available in the NCBI Sequence Read Archive (SRA) under BioProject PRJNA807936. Data corresponding to RNA-seq has been deposited under BioProject PRJNA824817.
- Bioinformatics scripts are available at https://github.com/UriFrankLab/Hsym_Hypostome_DiffExpr.
- Any additional information required to reanalyze the data reported in this paper is available from the lead contact upon request.

## Experimental Model and Study Participant Details

### Animals

Adult *Hydractinia symbiolongicarpus* colonies were maintained as described.^34^ Female and male colonies were grown on glass slides kept in artificial seawater (ASW) at 19-22ºC. Animals were fed four times per week with *Artemia* nauplii, and once a week with pureed oysters. To induce scheduled spawning, we kept the animals in a constant 14:10 light:dark cycle, where females and males spawn 1.5 hours after exposure to light.

## Method Details

### Hypostome isolation

One day starved adult *H. symbiolongicarpus* colonies were anaesthetized in 4% MgCl_2_ (in 50% distilled water / 50% filtered seawater). Polyps were dissected from the colony and decapitated. Hypostomes were isolated by removing the tentacles. Hypostomes were transferred to a glass Petri dish with freshly 0.2 *μ*m filtered seawater and incubated at 22.4-23°C with constant reciprocal shaking in a temperature-controlled incubator. 0.2 *μ*m filtered sea water was changed every two days.

### SpiDER-Gal staining

Hypostomes were collected and fixed in 0.2% glutaraldehyde (stock: 25%, Sigma-Aldrich; G5882), 4% paraformaldehyde (stock: 16%, Alfa Aesar; 43368) in filtered seawater for 2 minutes. Then, the fixative was replaced by 4% paraformaldehyde in filtered seawater and samples were incubated for 40 minutes. Samples were rinsed twice with seawater (3 minutes each) and proceed as per manufacturer’s indications. Samples were equilibrated with 1x Mcllvaine buffer, pH 6.0 (5x: 0.1 M citric acid solution, 0.2 M sodium phosphate solution) for 5 minutes. Then, samples were incubated with 1:500 SpiDER-Gal (1 mM; SG03-10, Dojindo Inc.) in 1x Mcllvaine buffer for 1 hour at RT. Samples were washed once with 1X PBST (0.3% TritonX-100 in 1X PBS) for 5 minutes, stained with Hoechst (20 mg/mL) 1:2000 in 1X PBST for 15 minutes, washed with 1X PBST, and mounted for imaging. Live animal staining with SpiDER-Gal was carried out in seawater.

### RNA Isolation from hypostome tissue

Total RNA was extracted from isolated hypostomes collected from wild type *H. symbiolongicarpus* strain 291-10. Samples consisted of three replicates of 220 hypostomes each at 0-, 1-, 3-, and 6-days post amputation (dpa). RNA was isolated using Direct-zol™ RNA MiniPrep kit (Zymo Research; R2050) per the manufacturer’s instructions. The eluted RNA was quantified using NanoDrop and the quality was checked using agarose-formaldehyde gel electrophoresis. After RNA isolation, samples were shipped to the NIH Intramural sequencing (NISC) for further processing and sequencing. RNA was amplified with the Ovation RNA-Seq System V2 kit, and sequencing libraries were made with Illumina TruSeq Stranded mRNA Library Prep Kit. Libraries were sequenced on one lane of Illumina NovaSeq 6000 (2x151 bp), generating between 87 and 137 million reads per sample (average 112 million reads). Only two replicates for 6 dpa were sequenced.

### Differential expression and candidate gene enrichment

Raw reads were trimmed and aligned to the reference genome using Trim Galore https://www.bioinformatics.babraham.ac.uk/projects/trim_galore/ and HISAT2^35^, respectively. A count matrix of mapped reads per genomic feature was generated using Subread featureCounts^36^ and converted into a DESeqDataSet. This dataset was used as an input to DESeq2^37^ in order to generate a list of differentially expressed genes for each time point compared to the subsequent time point. We used CellAgeDB to obtain a list of candidate genes involved in cellular senescence. We identified *Hydractinia* homologs of these genes from the genome by reciprocal BLAST search using query sequences obtained from Uniprot. We searched for these homologs in our list of differentially expressed genes in all time points and plotted a heatmap of differentially expressed genes, with the following parameters: Scale Type = None, Clustering Method = Average Linkage and Distance Measurement Method = Pearson.

### Transgenic animals

The generation of stable transgenic reporter animals was carried out as previously described^38^. Wnt3::GFP^8^ and RFamide::GFP^39^ reporters were previously generated in our lab. We used the GENEius online tool from Eurofins Genomics to codon optimize all the synthetic gene sequences for *Hydractinia symbiolongicarpus*. All synthetic genes were synthetized using IDT gBlocks gene fragments.

Fast Fluorescent timer protein reporter (FastFT): The codon optimized FastFT coding sequence^9^ was designed in frame with P2A peptide and membrane GFP sequences (FastFT-P2A-GFP^CAAX^). The synthetic sequence was amplified by PCR and inserted into the Piwi1 (or ß-tubulin) reporter plasmid^5^ using *NotI* and *SacI* restriction enzymes. G0 colonies were bred to G2 (non-mosaic transgenic offspring). The timer property of the FastFT fluorescent protein (earlier blue and later red fluorescent forms according to maturation state) was aimed to differentiate i-cells (recently translated protein) from their progeny. The mature red form was detected using an mCherry filter set. However, our microscopes lacked the proper filter setup to detect the FastFT blue form. Thus, to identify i-cells, we calibrated the laser power to higher GFP intensities^8^ that were devoid of or low in mCherry fluorescence (FastFT mature red form).

Cdki1 reporter: 5’ upstream and 3’ downstream regulatory sequences of the *Cdki1* gene (*HyS0010.253*) were cloned from extracted genomic DNA by PCR and inserted into an open cloning vector. Membrane GFP sequence (GFP^CAAX^) was placed in frame with the 5’ upstream regulatory sequence. G0 colonies were bred to G2 (non-mosaic transgenic offspring). OptoSOS: The OptoSos coding sequence^25^ was codon optimized and designed replacing the RFP sequence by our codon optimized mScarlet fluorescent protein^8^. The synthetic sequence was amplified by PCR and inserted into the ß-tubulin reporter construct^8^ using *NotI* and *SacI* restriction enzymes. We used BLAST to identify the endogenous *H. symbiolongicarpus* SOS catalytic domain that was amplified by PCR and inserted in frame with the SSPB sequence. Single injected embryos were grown to adult mosaic colonies, and we selected the animals who expressed the transgene in the hypostome. This procedure was repeated in *Cdki1* KO animals to generate the Cdki1 KO OptoSOS line from Figure 5.

### CRISPR/Cas9 knock-out

Single guide RNAs (sgRNAs) were designed using Geneious (2017.9.1.8) and CRISPRscan^40^ tools. The three selected sgRNAs targeting *Cdki1* did not match other genomic sequences. Modified synthetic sgRNAs were synthesized by Synthego Inc and diluted according to the manufacturer recommendations. To form the RNP complex with Cas9, the three sgRNAs were incubated together (500 ng/*μ*l total) with recombinant Cas9 (1*μ*g/*μ*l ; IDT, Cat. #1074181) for fifteen minutes at RT prior to being microinjected into zygotes.

### Single polyp genomic DNA extraction

Protocol was adapted from ref^8^. A single polyp was isolated from a colony and transferred to 1.5 ml Eppendorf tube. The polyp was suspended in 50 *μ*l Lysis buffer (10 mM Tris pH 8.0, 10 mM EDTA, 2% SDS), flicked few times, supplemented with 50 *μ*l of digestion buffer (10% SDS, 0.4 mg/ml Proteinase K), and incubated at 56°C for 2-3 hours with occasional flicking. Then, 100 *μ*l of cold phenol-chloroform (pH 8.0) was added. Samples were centrifuged at maximum speed for 15 minutes at 4°C, and supernatant was transferred to a new tube. Genomic DNA precipitation was carried out overnight at -20°C by adding two volumes of isopropanol and 1:10 total volume of Sodium Acetate Solution (3 M). Samples were cleaned up at 4°C by centrifugation and a 70% Ethanol wash. Extracted genomic DNA was dried out, resuspended in 10 *μ*l nuclease-free water, and stored for further genotyping PCR.

### Genotyping CRISPR/Cas9 knock-outs

One hundred CRISPR-Cas9 larvae that had been injected as zygotes were metamorphosed and grown into small colonies. Of these, forty animals were analyzed for *Cdki1* mutations. Primers spanning the entire coding region of the gene were used in a PCR reaction to identify large deletions. We kept and grew eight G0 mosaic colonies to sexual maturity and crossed one male with a wild-type female. One hundred G1 animals were metamorphosed, grown to small colonies, and genotyped. Two sets of primers were used to identify heterozygous colonies carrying the genomic deletion: one spanning the entire coding region and another internal primer targeting the deleted intron. PCR products of heterozygous colonies were cloned into PGEMT-easy vector and sequenced using the T7 and SP6 primers (File S3). Each of these animals was a heterozygote carrying a 3’ deletion mutation in one *Cdki1* allele at the predicted cut sites and one wild type allele. One male and one female were crossed and G2 offspring were genotyped identifying five mutant homozygous animals. We performed genotyping on these animals from two independent DNA extractions and confirmed the mutation by sequencing.

### SABER-FISH

Oligo probes were prepared according to^41^ using hairpins 27 and 30. Tissue was fixed and dehydrated as previously described in^8,39^: Tissue samples were incubated for 15 minutes in 4% MgCl2 (in 50% distilled water / 50% filtered seawater) and fixed in two steps. Fixative one: ice cold 0.2% glutaraldehyde (stock: 25%, Sigma-Aldrich; G5882), 4% paraformaldehyde (stock: 16%, Alfa Aesar; 43368) in filtered seawater for 90 seconds. Fixative two: 4% paraformaldehyde in in PBS-0.1% Tween (PTW) for one hour at 4°C. After three quick washes with PTW, samples were dehydrated in increasing concentrations of methanol in PTW and processed immediately by incubating in 1% H_2_O_2_ diluted in 100% MeOH (ice cold) for 45 min at 4°C. Followed by 2 quick washes with ice-cold 100% MeOH, samples were permeabilized in 75% acetone in 25% methanol and rehydrated into PTW. Two washes with glycine in PTW (2 mg/ml, 5min each; Fisher Scientific; BP381-1) followed by three PTW washes at RT. Samples were then washed three times with 1% (vol/vol) Triethanolamine pH 8.0 (TEA) in PTW for 5 min. 6 *μ*l and 12 *μ*l of acetic anhydride were added to the second and third TEA washes, respectively. Samples were then rinsed with PTW and incubated in pre-warmed Whyb buffer (2×SSC pH 7.0, 1% Tween-20, 40% Formamide) at 43°C for 10 min. Pre-hybridization and following steps were performed using pre-warmed reagents at 43°C according to ^41^. Whyb buffer was replaced with Hyb1 buffer (2×SSC pH 7.0, 1% Tween-20, 40% Formamide, 10% Dextran sulfate) and incubated at 43°C overnight (pre-hybridization). Samples were transfer to Hybe buffer (40% Formamide, 5x SSC, 0.05 mg/ml Heparin, 0.25% Tween20, 1% SDS, 1 mg/ml Salmon Sperm DNA, 1 mg/ml Roche blocking buffer powder) containing oligo probes at a concentration of 1 *μ*g/120*μ*l and hybridized for two days at 43°C. Probes were removed and replaced with Whyb buffer for 10 min, followed by two washes with Whyb buffer (30 min each), a 10 min wash with 50% Whyb buffer in 2x SSCTw (2×SSC pH 7.0, 0.1% Tween-20), and two washes with 2X SSCTw (10 min each). Then, 2x SSCTw was replaced with PTW (two quick washes). Samples were warmed up to 37°C, prewarmed Hyb2/fluor solution (1×PBS, 0.2% Tween-20, 10% Dextran sulfate/10 *μ*M Fluor Oligo) was added and incubated for one hour at 37°C. Hyb2/fluor solution was replaced with pre-warmed Whyb2 (1×PBS, 0.1% Tween-20, 30% Formamide) for a 10 min incubation at 37°C followed by two washes of 5 min each with PTW. Nuclear staining was then performed at RT by diluting Hoechst in PTW (1:2000) and incubating the samples for 45-60 min at RT. Samples were then quickly washed twice with PTW and mounted in 97% TDE. Samples were imaged within 4 days.

### Optogenetics

Isolated hypostomes from OptoSOS transgenic animals were exposed to 12 hours of constant blue illumination using a ReefLed™ 50 (RedSea) lamp. Exposure time, light intensities, and distance from the light source (6 cm) was determined experimentally to optimal survival rate. Higher intensity values and longer exposure times resulted in lethality. Two control experiments were carried out alongside the light induction: (1) OptoSOS isolated hypostomes under dark conditions, and (2) wild type hypostomes exposed to blue light. After the light induction period, samples were incubated without blue light until day 6 post amputation and fixed for immunofluorescence.

### Drug treatments

We incubated isolated hypostomes in 1 *μ*M of Rapamycin (Sigma-Aldrich; 553210) or 1 *μ*M of Navitoclax ABT-263 (MedChemExpress; HY-10087) for 28 hours. We determined this concentration empirically. Controls were incubated in equivalent concentrations of DMSO. Treatment time was determined experimentally to optimize survival rates. A portion of the samples was fix at 24 hours post amputation for SpiDER-Gal staining. After treatment, samples were rinsed with fresh seawater, incubated until day 6 post amputation, and fixed for Immunofluorescence.

### Cellular staining

Immunofluorescence (IF) staining was performed as previously described in^8^: the tissue was fixed in 4% Formaldehyde, 10 *μ*l/mL Acetic acid (glacial) in filtered seawater (FSW) for 90 minutes at RT, followed by three washes with 1X PBST (20 minutes each). PBST was replaced with 5% normal goat serum (NGS; diluted in PBST) and fixed samples were blocked for 1 to 2 hours at room temperature with gentle rocking. Primary antibodies were diluted in 5% NGS to desired concentration (anti-Piwi1 1:500; anti-mCherry 1:100). Blocking solution was removed, replaced with primary antibodies diluted in NGS, and incubated overnight at 4°C. Samples were washed three times with PBST (10 minutes each), secondary antibodies were then applied (1:250 in 5% NGS) and incubated for 2 hours at RT. Tissue was rinsed with PBST and stained with Hoechst (20 mg/ml) 1:2000 in 1X PBST for 15 minutes, washed with 1X PBST, and mounted for imaging.

### EdU staining

EdU staining was done as previously described in^5^. Isolated polyps and hypostomes were incubated in 0.01 mM EdU in FSW for 30 minutes, rinsed with FSW and incubated in MgCl_2_ before fixation. Hypostomes were incubated in EdU for 2 days (3-4 dpa). Samples were fixed in 4% Formaldehyde, 10 *μ*l/ml Acetic acid (glacial) in FSW for 90 minutes at room temperature (RT) and washed once with 3% BSA in PBST for 30min. Next, two washes with PBST for one hour and 30 minutes, respectively, followed by two washes with 3% BSA in PBST (5 minutes each). The tissue was then incubated in Click-iT cocktail for 30 minutes, followed by three washes of 3% BSA in PBST (20 minutes each).

### TUNEL labelling

TUNEL assays were performed using TM red In Situ Cell Death Detection Kit (#12156792910, Roche). Samples were fixed in 4% PFA in FSW for 90 minutes, then washed three times with PBS 0.01%Triton for 15 minutes, permeabilised with PBS 0.5%Triton for 90 minutes, and washed three times with 3% BSA in PBS for 15 minutes. Samples were incubated for 45 minutes at 37°C in a 50*μ*L mix composed of 25 *μ*L of reaction mix (Enzyme solution plus Label solution) and 25 *μ*l of 3% BSA in PBS. Negative controls were incubated in Label solution only and positive controls were incubated for 25 minutes at 37°C in DNAse I solution (Thermo Fisher Scientific, #EN0521) prior to incubation in reaction mix only. After the reaction, samples were washed in PBS and nuclei stained using Hoechst.

### Imaging

Confocal images for IF and SABER-FISH were collected using an inverted Olympus Fluoview 1000 and Olympus Fluoview 3000 laser scanning confocal microscopes.

For *in vivo* imaging, polyps from transgenic colonies were embedded in 0.8% low-melt agarose in filtered ASW and incubated in 35 mm imaging dishes with a glass bottom (Ibidi; D 263). Andor spinning disc and Olympus Fluoview 1000 laser scanning confocal microscopes were used to generate time-lapse movies and specific timepoint images. Raw images were visualized and analyzed using ImageJ/Fiji software and imaris viewer. Final figures were assembled using Adobe Illustrator and Adobe Photoshop.

*Piwi1* is expressed exclusively in i-cells. However, residual mRNA, protein, and the corresponding GFP reporter are present in decreasing concentrations in their progeny^8^. Hence, we calibrated the laser power to higher *Piwi1*^+^ intensity signals using an intact polyp. We repeated this procedure prior to every acquisition to discriminate between i-cells (higher intensities) and their progeny (lower intensities).

### Phylogenetic analysis

Multiple amino-acid alignments were generated with MAFFT 7 using default parameters. Gaps were removed using Gblocks 0.91b. Final alignments were composed of 1022, 155, and 174 amino acids for mTOR alignment, P53 family alignment, and CDKI family alignment, respectively. We used PhyML with maximum-likelihood method and 1000 bootstrap replicates. The best amino-acid evolution models to conduct analysis were determined, using MEGA11, to be the LG model for mTOR and P53 family alignment, and the JTT model for CDKI family alignment. Bayesian analyses were performed using MrBayes (v3.2.6) under mixed model. One fourth of the topologies were discarded (burn-in values), and the remaining ones were used to calculate the posterior probabilities. Bayesian analysis for mTOR, P53 family, and CDKI family ran for 200,000 generations with 5 randomly started simultaneous Markov chains (MC), 500,000 generations with 10 MC, and 3,000,000 with 15 MC, respectively. For all analyses, the first chain was a cold chain and the others were heated chains.

## Quantification and Statistical Analysis

Stained cells of fixed hypostomes were counted with the Fiji cell counter tool using the raw source data. Statistical analyses were executed using GraphPad prism software. We plotted the cell numbers of the different conditions and differences were assessed by comparing medians using Mann-Whitney U test.

## Supplementary Material

Fig S1

Fig S2

Fig S3

Fig S4

Fig S5

Fig S6

Fig S7

Fig S8

Fig S9

File S1 DE genes between day 1 vs day 0, day 3 vs day 1, and day 6 vs day 3. The file also includes candidate gene homologs in *Hydractinia* from CellAgeDB, and their differential expression.

File S2 Quantification analyses and statistics.

File S3

Video S1 Time lapse of an amputated *Cdki1*:GFPC^AAX^ reporter hypostome corresponding to Figure 3F.
