# Supplementary figures and images for: Senescence-induced cellular reprogramming drives cnidarian whole-body regeneration
Source: Cell Rep. Author manuscript; Available in PMC 2025 Mar 10. (PMC7617468; doi:10.1016/j.celrep.2023.112687)

**A**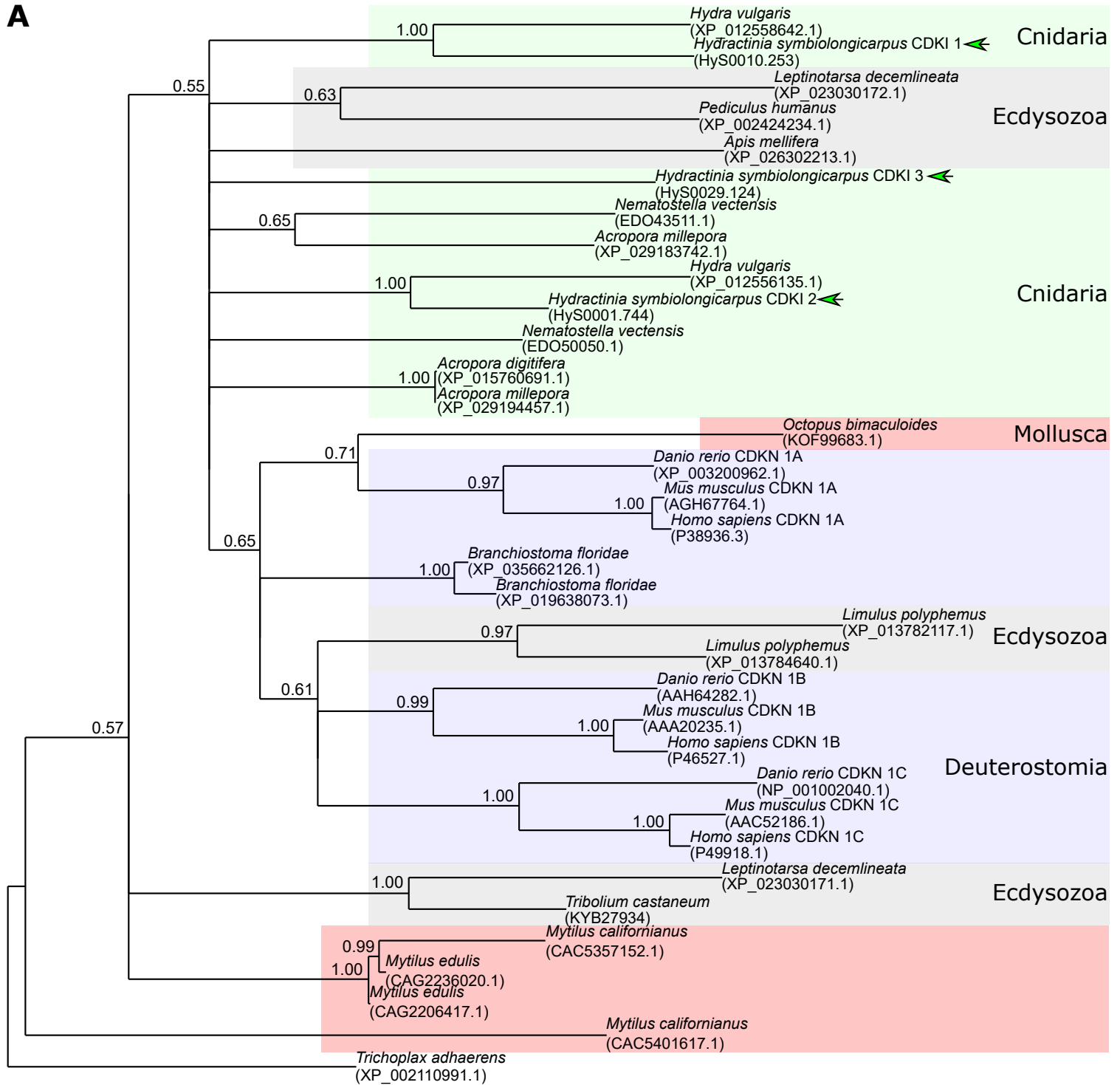

**B**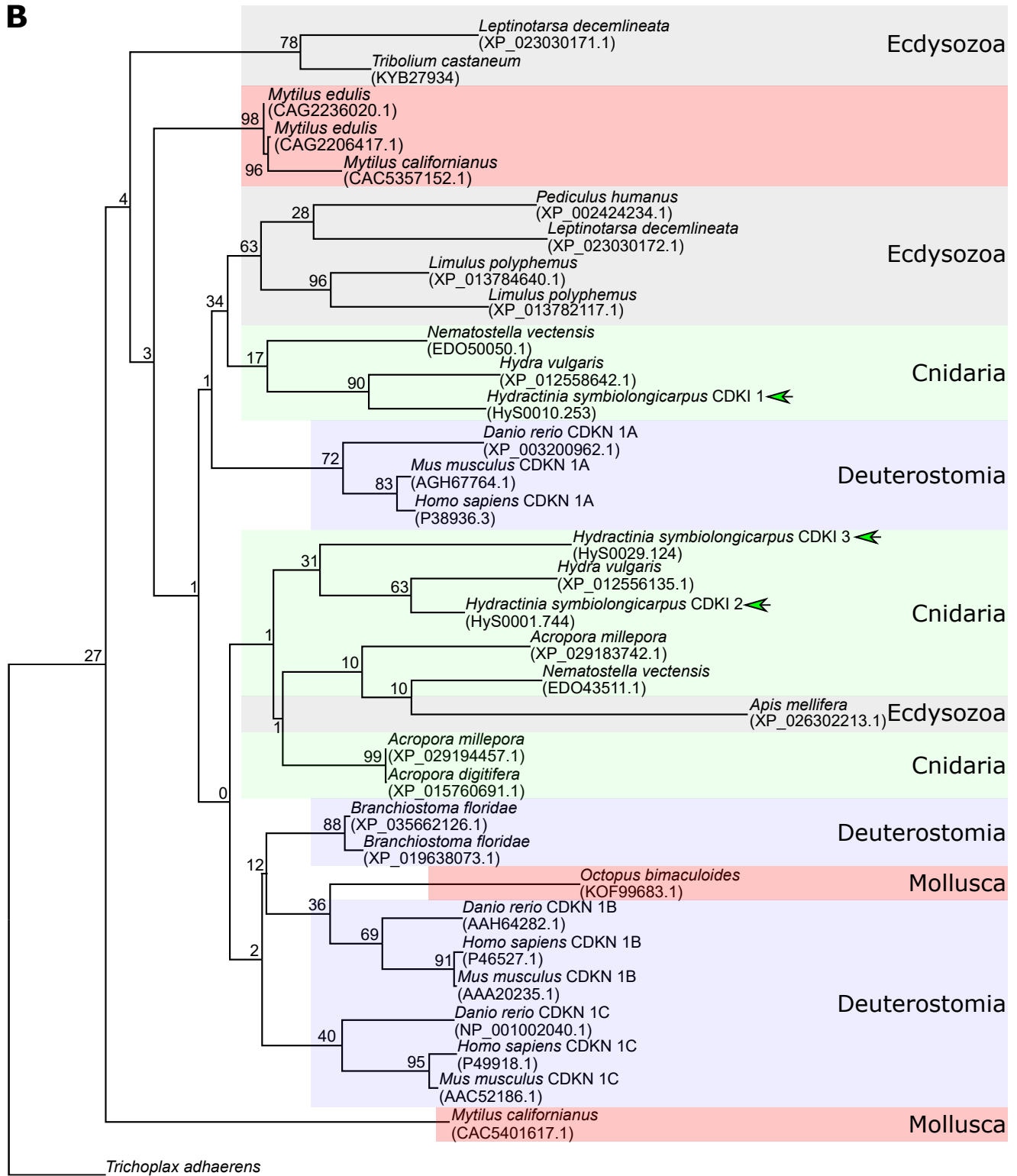

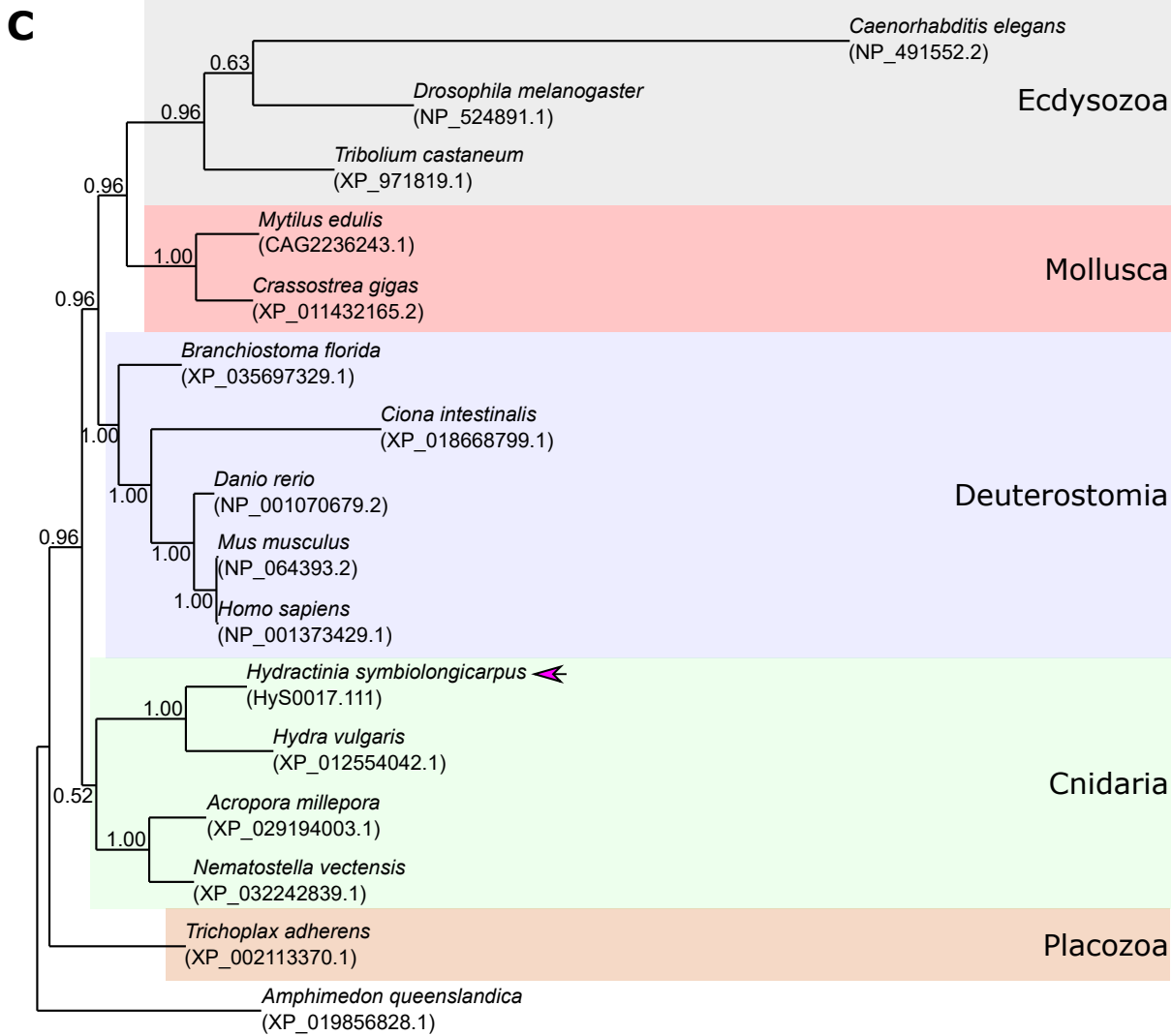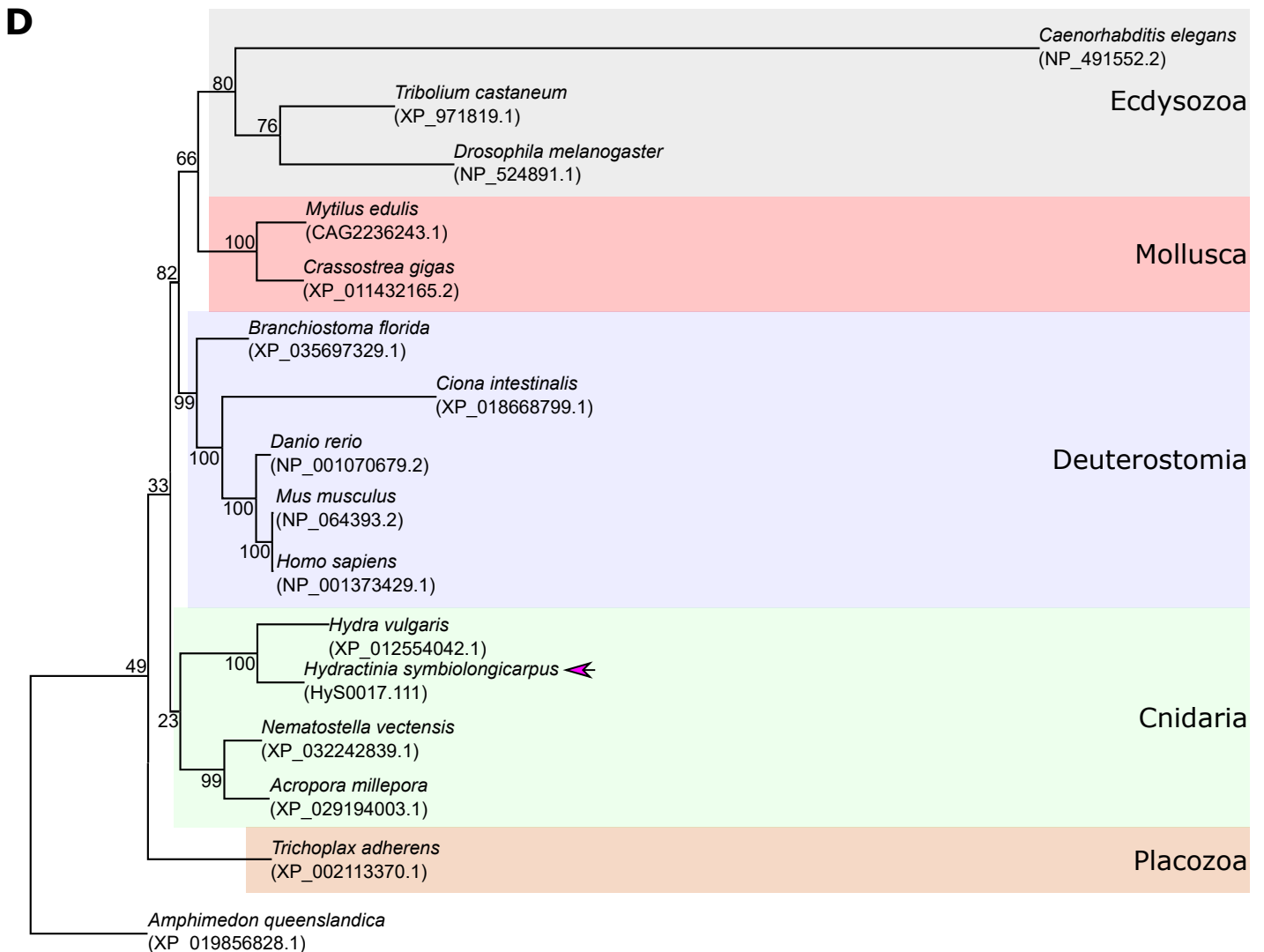

E

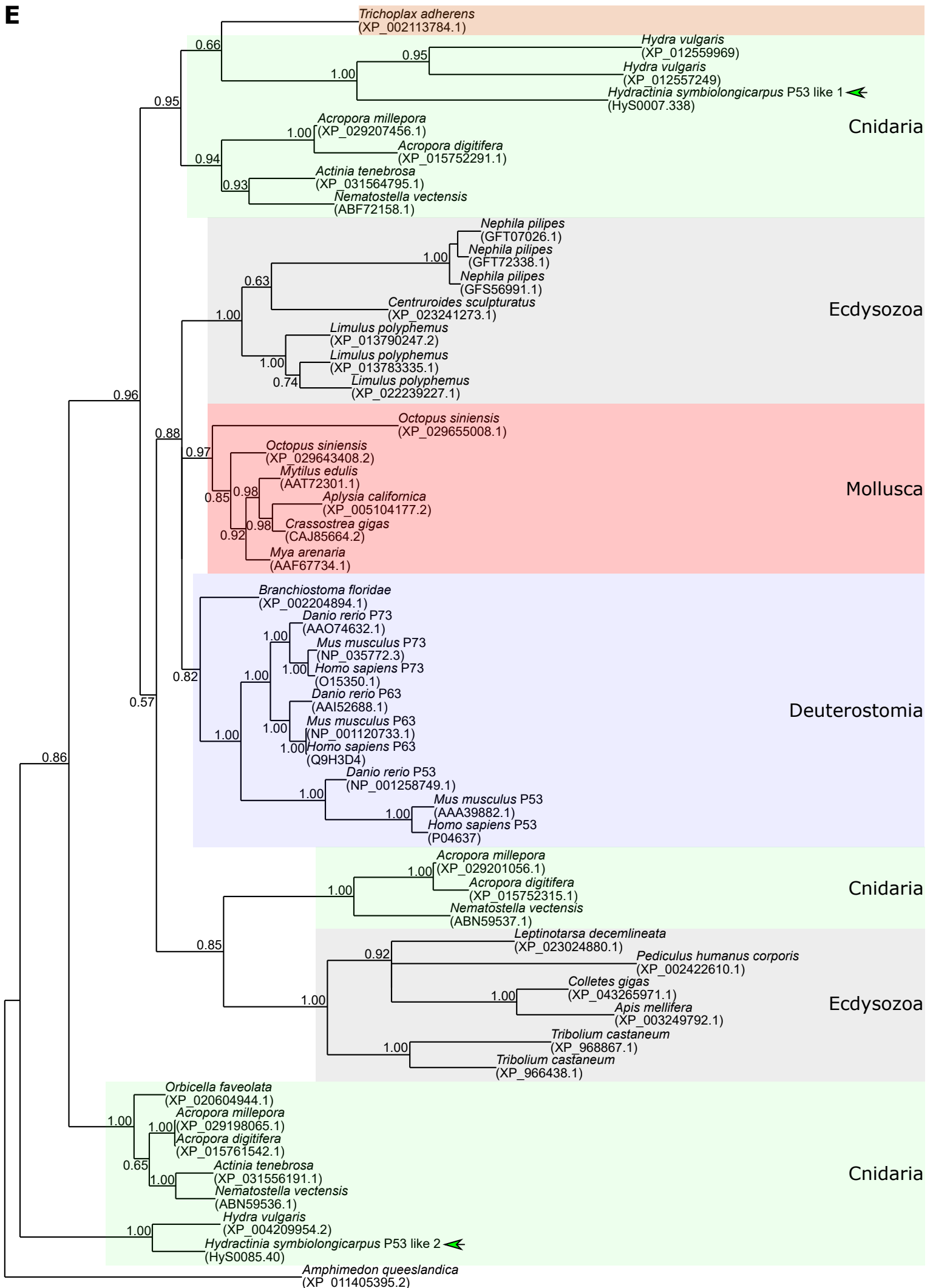

F

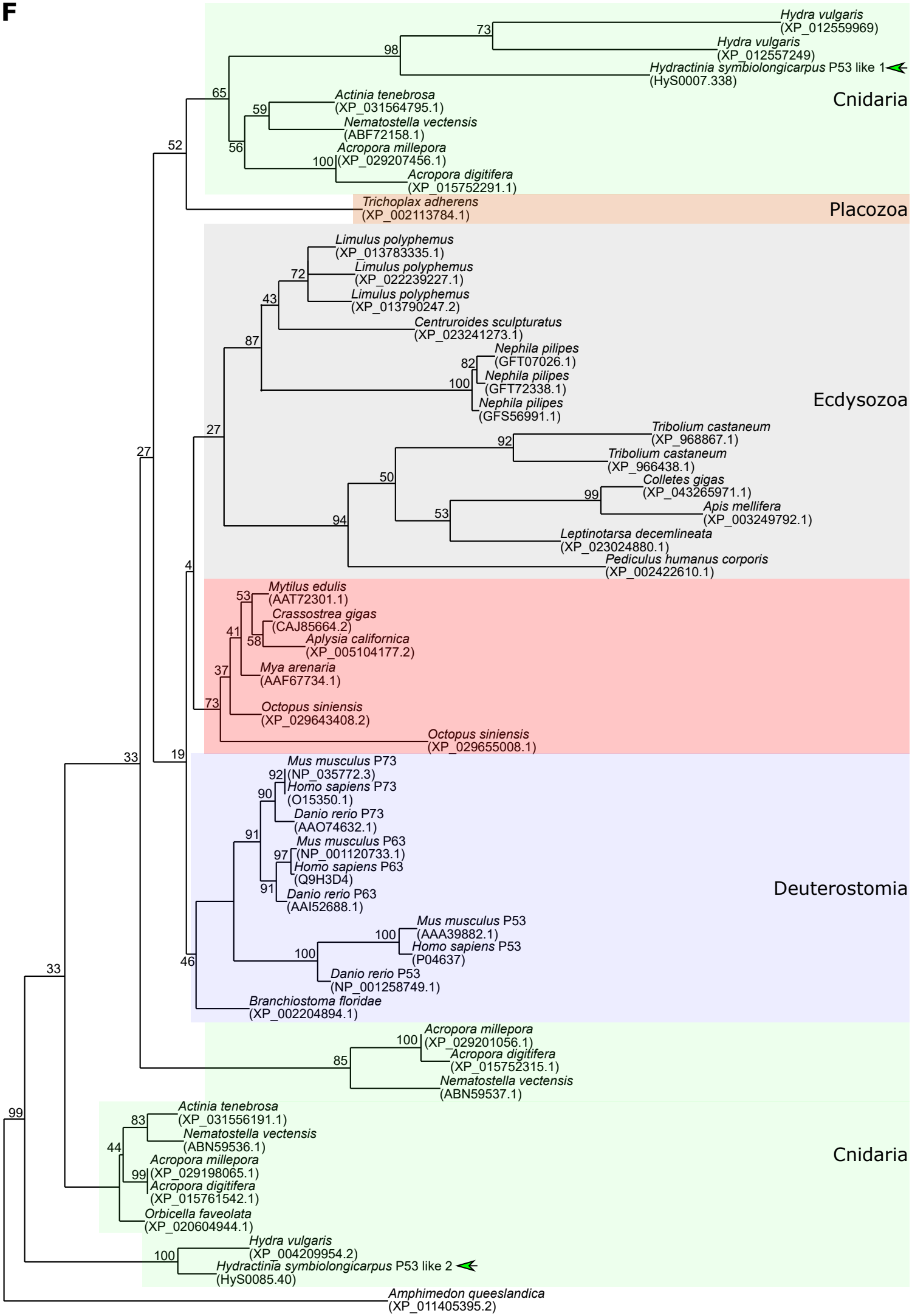

Supplement: Fig S4 [file EMS203328-supplement-Fig_S4.pdf]

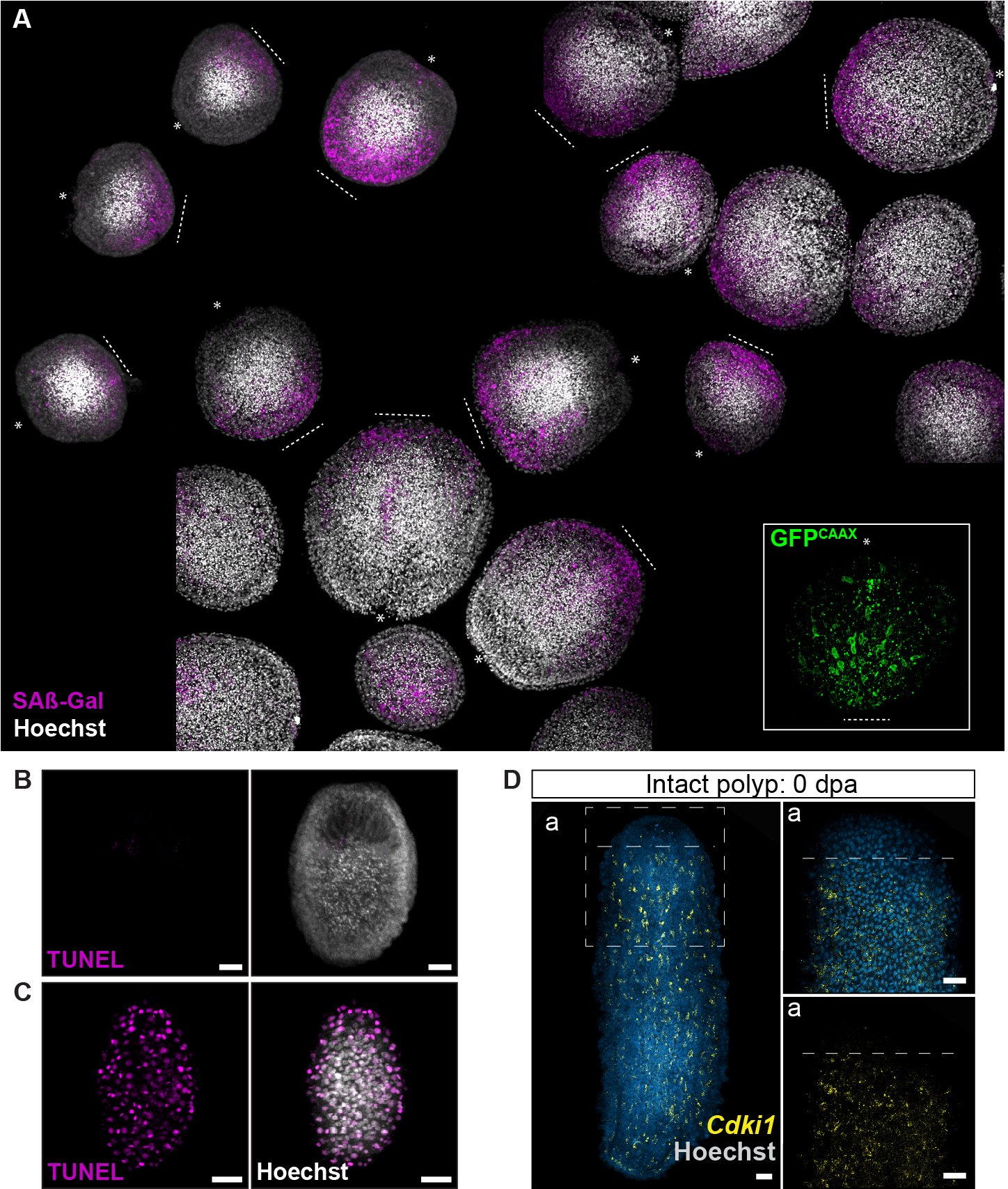

Supplement: Fig S6 [file EMS203328-supplement-Fig_S6.jpg]

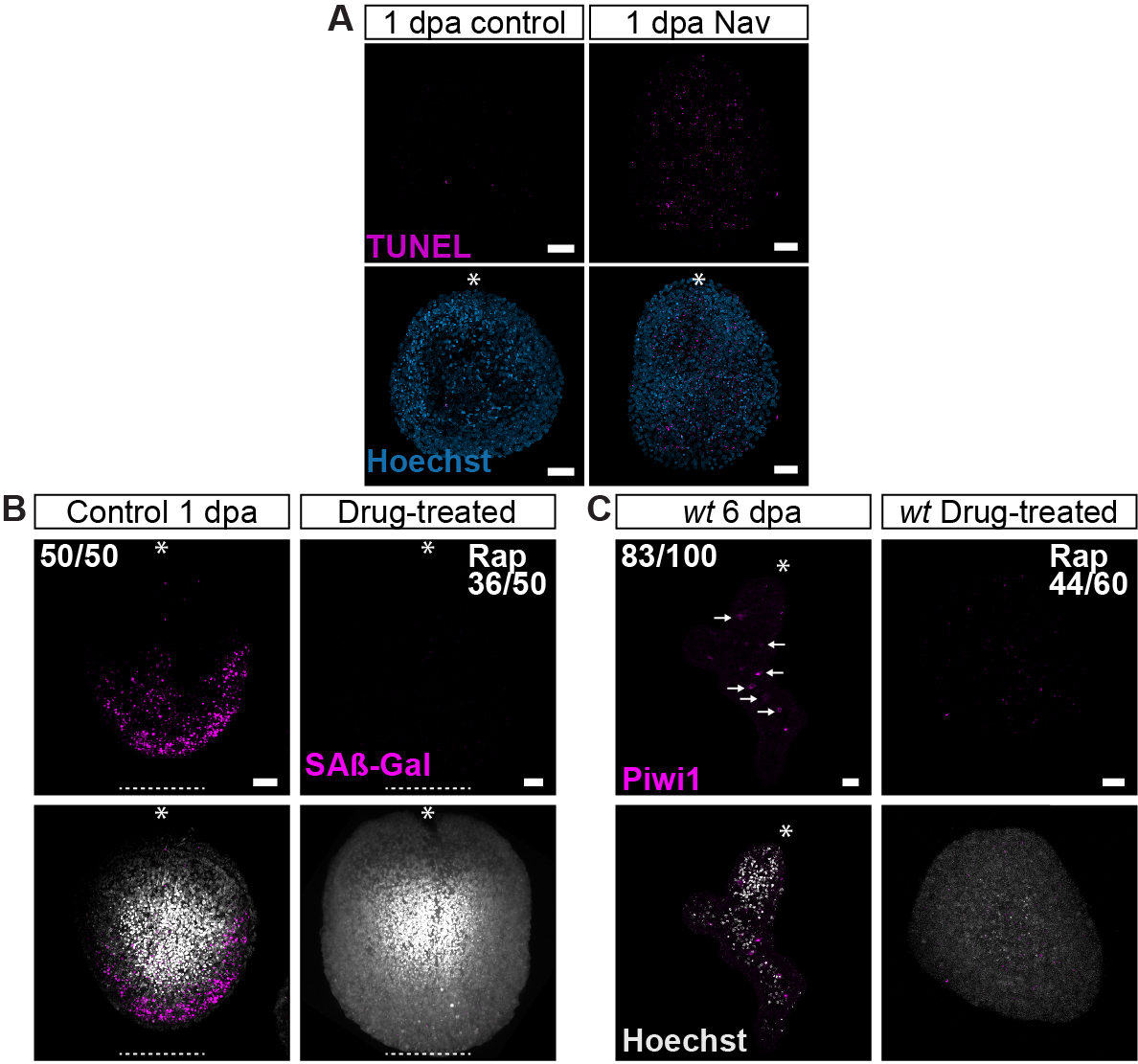

Supplement: Fig S7 [file EMS203328-supplement-Fig_S7.jpg]

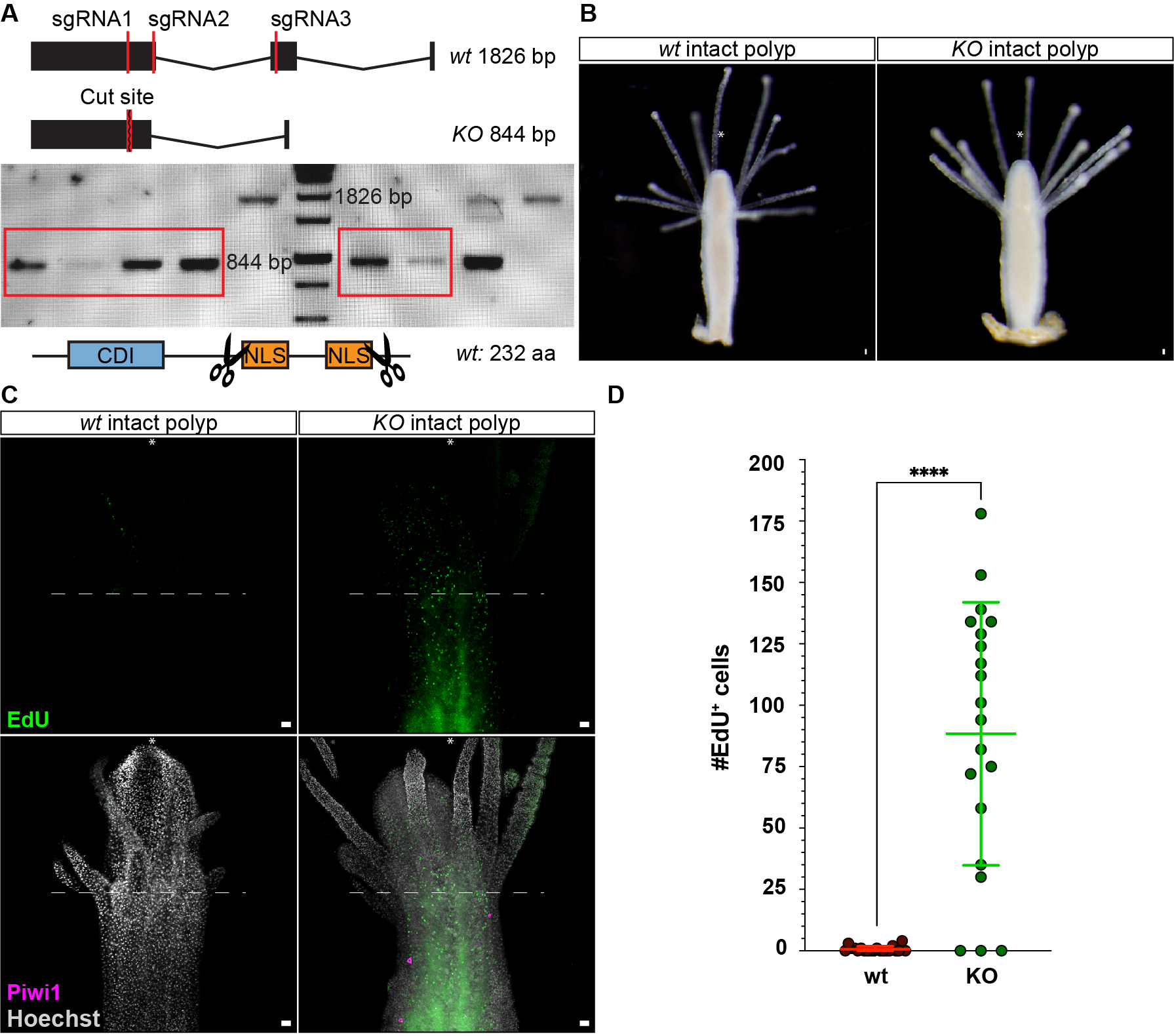

Supplement: Fig S8 [file EMS203328-supplement-Fig_S8.jpg]

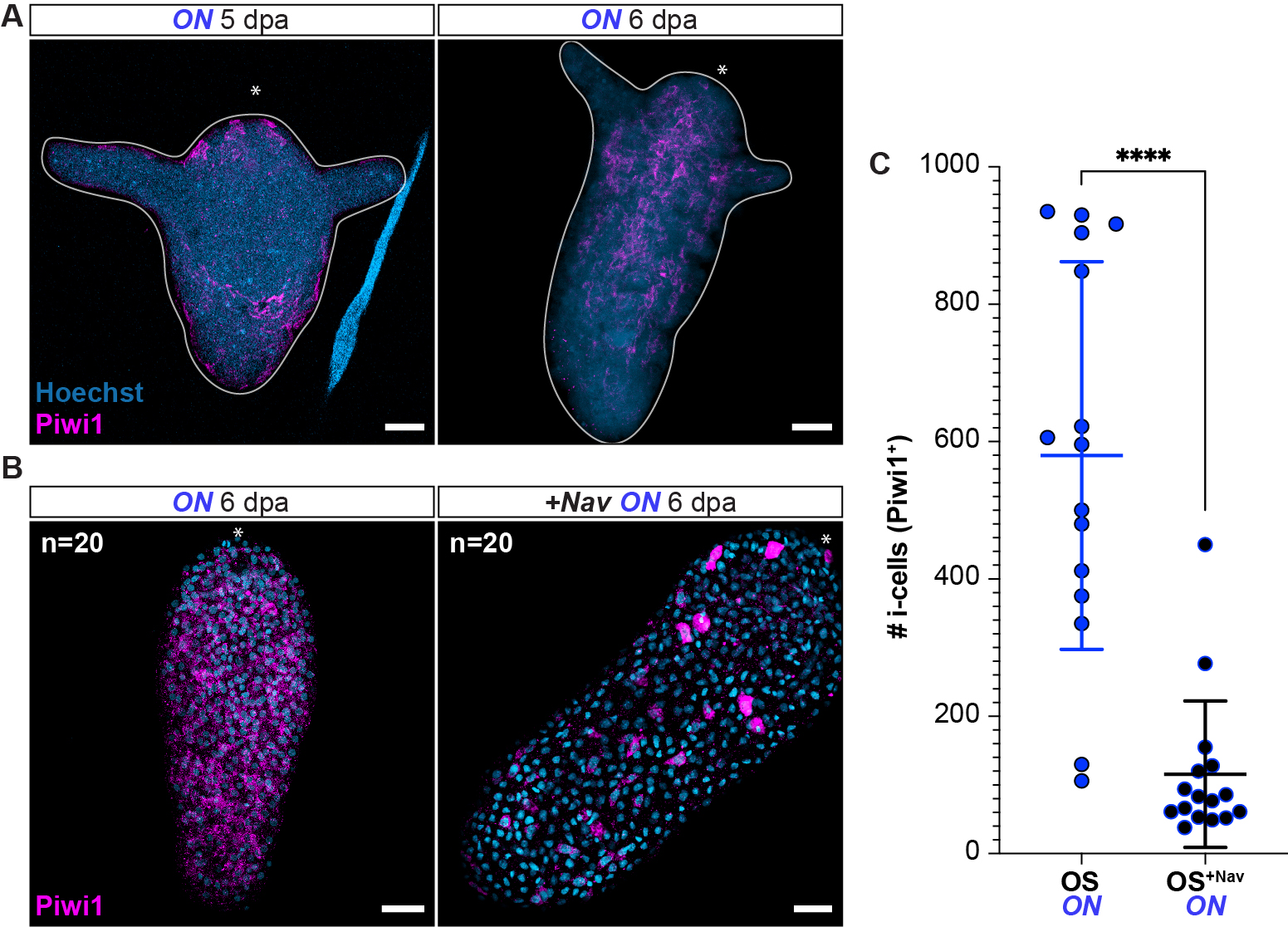

Supplement: Fig S9 [file EMS203328-supplement-Fig_S9.jpg]
