# Supplementary material for: Senescence-induced cellular reprogramming drives cnidarian whole-body regeneration
Source: Cell Rep. Author manuscript; Available in PMC 2025 Mar 10. (PMC7617468; doi:10.1016/j.celrep.2023.112687)
Supplement: File S3 [file EMS203328-supplement-File_S3.pdf]

Cdki1 CRISPR/Cas-9 wild type

5’**ATGGCACTTATAAGTGAATTGAGACGTTCTTTGTTCAATATTCCTGCTGGAAAAAAAGTTTGTCGTAGTTTATTTGGGAC**
TGTCGATCATGATGAAATAAAAGCAGACTTAAAAAAAGAATATAACGAACACATACAAATAAAGACAAAGTTATGGAAC
TTTGACTTTGAACGTAACATACCAAAGAATAAAGAAAATGGACTTAAATGGGTTGCTTGTCCAACTAAAGTAGCCGAGTA
TACTGTAAAGCCTGGAGTTAGTTTAATCACAGCATTTTTAGAAGAGGAAAAAGTCGCAAAAAATAGTTTGACGCATGAA
GAAAGACATCTTTCCTTGAGCCCAAATTCAGTTTCTGAAGGGTTAAGATCAAGACTTGAAGAGAATAATACTAAAGAAG
ATAAGAGCAAAGTGATTGACGAAGATTGTAGAATTGTACGTATTGTTAACACCTCCCCAACTCGTCCTCGGTTACATCGG
TCAAACCTGAACGACATTGTGACTCGTACAGTGCTTTCTACGACGAACATGTTATCCTGTGGTGGAAAAAGAAAACGAG
ATTCTCAGCCTCGTATTGATGGTAAGGAACTAAATTTTAAAATATTTCTAGCATTTTCAAGCTAAGACATAGGACGGTAGTTA
TTTCATTGTCTTCATAAAAAACTGTTATCTTCCATGACGTTAACTTTGATATGAAGGAGGGACTGATTTACCAGTATTTCTTTT
TTGTTATACTTCTGTTGCTAAGATGATATAATTTTTTTTATTAGAAAATTGGGACAGGCCCGCCTCACTATTTGATGTTTGTAC
CGATGAGTACACAACTTTTTCCTAAATGAATTTTTTTTATTGTTTAAAATTTCTTTGTTTGGTGAAGAAAAATATTGCTACCCT
ATTAATTATTGATAACGCGTGGAGGCACATATGTCTGAGCTCTGTGATAAAACATGATGCCGAGGTGTGTAAATATCGGCG
TCTATACTGAACCTTACACCTGGGAGTGCAAGAAAATTATTATACTTACACCGCTCATTAATTTGAATGTTTTATCTATTATAC
TTAATGATTTGGCTTATAATATCTATATATTTTATTTTATAATTAACTAGGTATTAAAAGCTTAAAATAATTTCTAAAGGCTAGT
GTCTTTCCCCATTGTGTAGAGTGGTTGATTAGCAAAACATTGGGGTCCAAAAGTTTAGCATTATTAATTTTAGTGAACCTTTC
CTTGTTGTACATAACAACACGTGTTCTTAGGAAATACTTCTTATTTTCTGTTTATATAAATAATTCAATGTATATATCATGAAG
TAGAGTTTTGTAAAAAATGACTCAGCGATTTCTATCTCGAACAAAGAAAACTCCAAAAAAGAAGAAACCTGATAAGTATTAAA
TTTTAATATTTTTCTTCGTTTAG**ATTTTATGAAGAAAAAGAAACCTCGCGTATCAATGGATCTATCCCAGAAATCTTTACGA**
AAAGATATCTCTCCTATTTCAAGACGACTACGGTCATCACCCTCAACTCAGGTATGATGAACCTTTTTTATTGACAAATTCA
AAAGTCATTACATATTTTCCTGAAAAAAAATAACTATTTCCGAATCCAATTTAAAACGCCGGTTTGCTGGGTGACATTTATAC
TATATTGACGTTTCTCATTCATTGTATGTTCGCATACGCTTCAATGAATGTTTAATTACTACGATGTAGTGCATCTGGCTATC
TGTAGTATCGCCATAGCAGCCGCCATTTTGAAGAGACGTTTGTTTTTGTTTTTATTCACTTTTTTATTTATTTGTTTATTTTGTT
GGTTTTAGGT**TTCCGTATAG**3’

Exons – Fwd1, Rev1 – Fwd2, Rev2 – sgRNAs

Cdki1 CRISPR/Cas-9 Knockout

5’ATGGCACTTATAAGTGAATTGAGACGTTCTTTGTTCAATATTCCTGCTGGAAAAAAAGTTTGTCGTAGTTTATTTGGGAC
TGTCGATCATGATGAAATAAAAGCAGACTTAAAAAAAGAATATAACGAACACATACAAATAAAGACAAAGTTATGGAAC
TTTGACTTTGAACGTAACATACCAAAGAATAAAGAAAATGGACTTAAATGGGTTGCTTGTCCAACTAAAGTAGCCGAGTA
TACTGTAAAGCCTGGAGTTAGTTTAATCACAGCATTTTTAGAAGAGGAAAAAGTCGCAAAAAATAGTTTGACGCATGAA
GAAAGACATCTTTCCTTGAGCCCAAATTCAGTTTCTGAAGGGTTAAGATCAAGACTTGAAGAGAATAATACTAAAGAAG
ATAAGAGCAAAGTGATTGACGAAGATTGTAGAATTGTACGTATTGTTAACACCTCCCCAAC/GTATCAATGGATCTATCC
CAGAAATCTTTACGAAAAGATATCTCTCCTATTTCAAGACGACTACGGTCATCACCCTCAACTCAGGTATGATGAACCTTT
TTTATTGACAAATTCAAAAGTCATTACATATTTTCCTGAAAAAAAATAACTATTTCCGAATCCAATTTAAAACGCCGGTTTGCT
GGGTGACATTTATACTATATTGACGTTTCTCATTCATTGTATGTTCGCATACGCTTCAATGAATGTTTAATTACTACGATGTA
GTGCATCTGGCTATCTGTAGTATCGCCATAGCAGCCGCCATTTTGAAGAGACGTTTGTTTTTGTTTTTATTCACTTTTTTATT
TATTTGTTTATTTTGTTGGTTTTAGGT**TTCCGTATAG**3’

Exons (precut) – Fwd1, Rev1 – sgRNAs/cut sites

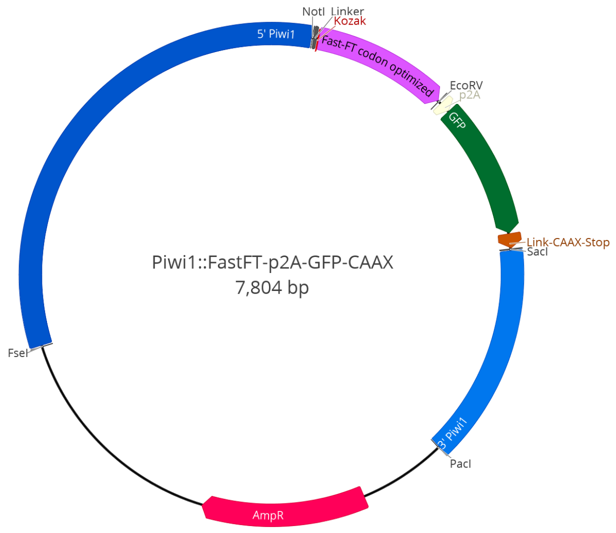

GGCCGGCCATT==**5’Piwi1**=CAGATGATCCGCAGACAATAGACCTTTTTGAATGTTTACATTTTCCGCGGTAAGCCCTGTCAG
CTTTAACAATTGCATGGCAGGCAAAAAGATGCGGGTGCTAGGTAAAGTGAGGTCCTTTTTAGTCTGTTTTATTATTACCACT
TTATATGAATATATGTCAATCAAAAGTTATCTATCTACACTCGATAATAGTTTAAAGCCTCAAAAATGTTTCCGTAGAAAGAAA
TGGGCTGAAAAAGACCTTTCTTCAGCAAGCTTCCGCATCTTTCTGCCTGCCACGCAGTTGTTAGAGCCAGAAGGGCTACAC
GAAAATGTAAACATTTAAAAAGGTCTATAGACATGTTTAATTTGCCCTAATTTTTGGTTGGCAATAGAATGAATATATATATAA
GGAATAATGAAATTTTCCCCCATGTATTATTTAATATGTCTAGGACAAAAAAGTCATGTGACTTGGCTTTTAGCCAATAAGAT
TGTTGTAATTTTTAGGGGGAAAACACTGTGGCAAAATGTTCCTATAACACTTTTATATGCAACTCATATCATAAATTCAGGAG
AGTGTGTATTGTTTGCCCCAAATGTGATGTAATCATTGCCACACAAGAATACAATATCTAGCTGGCAGGATCCCTTGTTTGC
TGCTTGACCTCAACTGTTATATGTGGATGTTTGCAGATTATCAGCACCCTTGTCTCATAATGAAAAGTGAAAAAATCCCCGC
CAACCCTCTAGATAGATAAGGAATACATGAATTCACAGTTTCAGCTAACTATTGTCTAAATAAATATTTCTTTTGTTACAATGC
TTTTTTTGAGAATTTAAATAAAGGAAGTGAACTGTTATCAAGAAGGCTCTGGCCATGAAGAAAAGTGAAATAATTTTTTTGTG
AAATATCTTGAAAAAAAAAAGTTCAAATTCCTTCACATTGTTTTAAGTAGGGTTACTGCCAATCTATGAAGATTGGTCAGTGGT
CCCTCGCTTTACATGAAAAAATAGCCAGACTTCAACGCCAAGTATAAACCACTCTGCCTGCTACACATATAGGCTATTCACC
TAGATATAGTCATGCAGCCTTGGCAGCGATTCAATATGGGCAATTGAATATTTAGCATAGCCCCCGGGTTAACTCAAGCCA
TGTGAGAGAATTGGGGAGATAGCGCACTGTGTGGTGGGCCGTTGGATGTTGCAGGGAGTTGTCTGGCAGAGCGCTGACC
CTAATTGAGTTTACGTAGCTCAAAGAGAGCATTAAATACTCCAGGATAGATAGATAGATAGATCTACTATCTATCTATCTAGC
TCAAAGAGAGCATTAAATACTCCAGGACTCCCCATCAAAGCCATGACCCCTCCTGGAAATAATAGACAGGGTATATCCCTC
GATATTTGTGAGGCTAGCCATGTAAAATATGCACATCTATCTATCTATTACATGATAACTGGTCTGGTCTTGGTACACATGC
ATCACCACTTTCCCTACCATGTTTTATTTTGTGATGCTGCGCCAAAGTATATCACAATGGTTGTATTTGTTCAAATTCAGCGC
CCCTTTAGCTTACGGTGGATTTTCACTTTGATTTCAGTGAGGAACAGAAATGTGCAAGGTTACCGCGTTATATGATATTTATT
TTTTATTCATTTTTTTTATCCCATCCTTTTTTAACTTCTAGCACAAAATAATTTTTAGTCTCTTTAAAAATTATCGCACATTAAAT
GTTGAATTAAAGATTCATAAAAACTTTTTTTTTAGTGTGATAATTTATATTATATACCACAAGTTTTGTTTGTTGTCCACTTATT
GACTAAAGTCATAATAGTCACTCGGAATATGACTAAAATTAAAATAAATGCATGTTTTCTTATTGTCGTATTTTCAAAAAATAT
AAATTTTTATCTGTCTCCCCTCCTATTCTTTTCCTTTCAAACAAAACTTGACAAGTGGAAACCCACCCTTGTGTGAAGCAAGG
TTGGTTTCTGTTTGGTGCATTATTAGACCATCACTGAACTTCTTCTCAACATTTATGAATAAAATAACGTATTTTGAGTCATAT
TTAATAATTACGAAAATTTTCATTACATTTATTTAACATTTTCTTGAAATGCCTATTTTACCAATCACCAAAGAATTTTTTGGAT
TTGGCTTTAATGCAACATGTAAAAACCACGCTACAATGATTTTCAACCAATACGGTCGACATGCATAAATTAGCATAATAATT
ACGCCTCATGGTTCTAGTTGATGAAAGTTCGTTCGGAGAGTAGACGCGTTTAGGGTAAGAAAGAAAGTACCTGTTTTGTTA
TTAATACGTGGAAGAAAAAGGTTGCCAAGGGGTAATATAATACCCAGGAGATTAATAAAGATAGTCTTACGAGTTATAAGTT
AAAACTAATCGGAAATCTCTAGAATTATCACTTTCACTGTTTTTGTCAGCTAGCTATAGCTATTATTTATATGTAGTAGGTTTT
GAGGTAGGCTACAACTAGCTAAAGTATCTTCCTGTTATTAAGTTTTTAGAAAAATTATAAGAAGAAAACGCGGCCGCGGTGG
AGGTGGATCTGAAAAAGCCACC==**FastFT**=ATGGTCAGCAAAGGCGAAGAAGACAATATGGCGATAATCAAGGAATTTATG
CGCTTTAAAGTACACGTAGAAGGAAGTGTAAATGGGCATGAATTCGAAATTGAAGGAGAGGGAAAGGGTCGGCCCTACGA
AGGCACGCAGACAGCCAAGTTAAAAGTGACCAAAGGAGGCCCGTTACCATTTGCATGGGACATACTCTCACCACAATTCAT
GTACGGATCCCGAGCCTATGTCAAGCATCCTGCTGATATACCAGACTATTGGAAACTTAGTTTCCCGGAGGGATTTAAGTG
GGAGAGAGTCATGAACTTTGAGGATGGAGGTGTTGTGACCGTCACACAAGACTCCTCTCTGCAGGATGGTGAGTTTATTTA
TAAAGTTAAATTGCGAGGAACAAATTTTCCTTCAGATGGACCAGTTATGCAAAAAAAAACTATGGGTTGGGAAGCTTCAACT
GAAAGGATGTACCCTGAAGATGGTGCTCTAAAGGGAGAAATCAAACAACGTTTAAAATTGAAGGATGGTGGTCACTACGAT
GCAGAAGTTAAAACTACATATAAAGCAAAGAAACCAGTTCAATTACCAGGTGCATATAACGTTAACATCAAACTTGATATTAC
ATCGCATAACGAAGATTACACAATTGTTGAACAATATGAAAGATCGGAAGGTAGACACTCTACAGGAGGTATGGATGAATT
GTACAAAGATATCGGTTCAGGT==**P2A**=GCTACAAATTTTTCATTATTAAAACAAGCTGGTGATGTTGAAGAAAATCCAGGTC
CA==**GFP**^{CAAX}=ATGAGTAAAGGAGAAGAACTTTTCACTGGAGTTGTCCCAATTCTTGTTGAATTAGATGGTGATGTTAATGGG
CACAAATTTTCTGTCAGTGGAGAGGGTGAAGGTGATGCAACATACGGAAAACTTACCCTTAAATTTATTTGCACTACTGGAA
AACTACCTGTTCCATGGCCAACACTTGTCACTACTTTCTGTTATGGTGTTCAATGCTTTTCAAGATACCCAGATCATATGAAA
CGGCATGACTTTTTCAAGAGTGCCATGCCCGAAGGTTATGTACAGGAAAGAACTATATTTTTCAAAGATGACGGGAACTAC
AAGACACGTGCTGAAGTCAAGTTTGAAGGTGATACCCTTGTTAATAGAATCGAGTTAAAAGGTATTGATTTTAAAGAAGATG
GAAACATTCTTGGACACAAATTGGAATACAACTATAACTCACACAATGTATACATCATGGCAGACAAACAAAAGAATGGAAT
CAAAGTTAACTTCAAAATTAGACACAACATTGAAGATGGAAGCGTTCAACTAGCAGACCATTATCAACAAAATACTCCAATT
GGCGATGGCCCTGTCCTTTTACCAGACAACCATTACCTGTCCACACAATCTGCCCTTTCGAAAGATCCCAACGAAAAGAGA

GACCACATGGTCCTTCTTGAGTTTGTAACAGCTGCTGGGATTACACATGGCATGGATGAACTATACAAAAGTGGACTTAGA
TCAAAATTGAACCCCCCGGACGAATCGGGTCCTGGTTGCATGAGTTGCAAATGTGTATTATCATGAATGAGCTC==**3’Piwi1**=
GTAGCTGCGCGTTGTTTACGTGGAGGTTGTCTTGCGCTATGACTTCTAAAAACTGAAAAGTATTGTTTATATTGTTTATATTG
GCGTTTATATTTTTAACAAATAACCAGTAGTAGTAGTAGTATTTTATTTTAAAACGATTCATATAGGTTGTATTTTCTTGTGTTT
GAGACTTTTTTTTAGCTATATAAGCTTGTTCTTGGGTTATTTTTGACATAGTTTACAAAGATTGCTTACAATCTTACCTTTATG
TTCATGCAGTGTCGTCAAGATATTCACCTTAGAAGTAGCAGTTGCTTGTTGAATCATTTTTTCCCTAATCATTTTCACGAAGA
GCAAATGTAAAGAGTTTACAAATCTGTTTTAGATTTCGTTTTGAGATGCGGGTGTAAAAAATGTTTTTAGCAAAGGATTTTTG
GTCCATTTTTATATTTTTGTTCATTTTCATATTTTTTTCGAGAAAAGTGATCACACTAAAGAGATGTAGTCGTTGTTCATCTGC
AGACCTAGTGTTATCTGTATATCCAGCAAGCGACGGTTAAAATCCGATATTCGCTCTGCAGCATACCGCGTGACGAAGCTA
TCTATTCAACGGCCGGTGCGCATTCTAACGTCACCAAATCTTTTAGTTATGCAATAGGTGAAAGTTACTGTTTTGGGGTTAT
TTTTACGACTAAATCAGGTCTAGGAAAATAATGGTAGCAAATAAATTTTTAAAAAAATGTCTATGCGAAAATAATAAAATAGC
CAACGCAGTTAATTTTCTTTTGCATCAATAGTATTTCCTAAAAGAACGTAAAAACATGGTTTGCAACGTTGTTTCAAACAGTC
GAACGCTGTATTGAGATACTTAGGCTGTCTTATCAAGAGGAAAGGGTGGTGGAAAACATCCTATTTCTCCTCCGTGCGCTT
TATCCAGAAACGTAATGCACGCGATGGGACTACTTTCGCCACAATATAAAGGCCGCTGCTCATTGAGACGTTCTTAAGCCT
AGTGCTCATTTATCGTCTTAGGCTTACGTTGAAGCAGTCTTAGGCTAAGGCGAATATAAAAAAAATTCAGATCTTAAGCCGT
ATGTCAAATAAGTGTAAACGCTAATTCACACTAGCGTAAGCCTTCTTAATTAACGTACGGGCCCTTTCGTCTCGCGCGTTTC
GGTGATGACGGTGAAAACCTCTGACACATGCAGCTCCCGGAGACGGTCACAGCTTGTCTGTAAGCGGATGCCGGGAGCA
GACAAGCCCGTCAGGGCGCGTCAGCGGGTGTTGGCGGGTGTCGGGGCTGGCTTAACTATGCGGCATCAGAGCCGATTG
TACTGAGAGTGCACCATATGCGGTGTGAAATACCGCACAGATGCGTAAGGAGAAAATACCGCATCAGGCGGCCTTAAGGG
CCTCGTGATACGCCTATTTTTATAGGTTAATGTCATGATAATAATGGTTTCTTAGACGTCAGGTGGCACTTTTCGGGGAAAT
GTGCGCGGAACCCCTATTTGTTTATTTTTCTAAATACATTCAAATATGTATCCGCTCATGAGACAATAACCCTGATAAATGCT
TCAATAATATTGAAAAAGGAAGAGTATGAGTATTCAACATTTCCGTGTCGCCCTTATTCCCTTTTTTGCGGCATTTTGCCTTC
CTGTTTTTGCTCACCCAGAAACGCTGGTGAAAGTAAAAGATGCTGAAGATCAGTTGGGTGCACGAGTGGGTTACATCGAAC
TGGATCTCAACAGCGGTAAGATCCTTGAGAGTTTTCGCCCCGAAGAACGTTTTCCAATGATGAGCACTTTTAAAGTTCTGCT
ATGTGGCGCGGTATTATCCCGTATTGACGCCGGGCAAGAGCAACTCGGTCGCCGCATACACTATTCTCAGAATGACTTGG
TTGAGTACTCACCAGTCACAGAAAAGCATCTTACGGATGGCATGACAGTAAGAGAATTATGCAGTGCTGCCATAACCATGA
GTGATAACACTGCGGCCAACTTACTTCTGACAACGATCGGAGGACCGAAGGAGCTAACCGCTTTTTTGCACAACATGGGG
GATCATGTAACTCGCCTTGATCGTTGGGAACCGGAGCTGAATGAAGCCATACCAAACGACGAGCGTGACACCACGATGCC
TGTAGCAATGGCAACAACGTTGCGCAAACTATTAACTGGCGAACTACTTACTCTAGCTTCCCGGCAACAATTAATAGACTG
GATGGAGGCGGATAAAGTTGCAGGACCACTTCTGCGCTCGGCCCTTCCGGCTGGCTGGTTTATTGCTGATAAATCTGGAG
CCGGTGAGCGTGGGTCTCGCGGTATCATTGCAGCACTGGGGCCAGATGGTAAGCCCTCCCGTATCGTAGTTATCTACACG
ACGGGGAGTCAGGCAACTATGGATGAACGAAATAGACAGATCGCTGAGATAGGTGCCTCACTGATTAAGCATTGGTAACT
GTCAGACCAAGTTTACTCATATATACTTTAGATTGATTTAAAACTTCATTTTTAATTTAAAAGGATCTAGGTGAAGATCCTTTT
TGATAATCTCATGACCAAAATCCCTTAACGTGAGTTTTCGTTCCACTGAGCGTCAGACCCCGTAGAAAAGATCAAAGGATCT
TCTTGAGATCCTTTTTTTCTGCGCGTAATCTGCTGCTTGCAAACAAAAAAACCACCGCTACCAGCGGTGGTTTGTTTGCCG
GATCAAGAGCTACCAACTCTTTTTCCGAAGGTAACTGGCTTCAGCAGAGCGCAGATACCAAATACTGTCCTTCTAGTGTAG
CCGTAGTTAGGCCACCACTTCAAGAACTCTGTAGCACCGCCTACATACCTCGCTCTGCTAATCCTGTTACCAGTGGCTGCT
GCCAGTGGCGATAAGTCGTGTCTTACCGGGTTGGACTCAAGACGATAGTTACCGGATAAGGCGCAGCGGTCGGGCTGAA
CGGGGGGTTCGTGCACACAGCCCAGCTTGGAGCGAACGACCTACACCGAACTGAGATACCTACAGCGTGAGCTATGAGA
AAGCGCCACGCTTCCCGAAGGGAGAAAGGCGGACAGGTATCCGGTAAGCGGCAGGGTCGGAACAGGAGAGCGCACGAG
GGAGCTTCCAGGGGGAAACGCCTGGTATCTTTATAGTCCTGTCGGGTTTCGCCACCTCTGACTTGAGCGTCGATTTTTGTG
ATGCTCGTCAGGGGGGCGGAGCCTATGGAAAAACGCCAGCAACGCGGCCTTTTTACGGTTCCTGGCCTTTTGCTGGCCTT
TTGCTCACATGTTCTTTCCTGCGTTATCCCCTGATTCTGTGGATAACCGTATTACCGCCTTTGAGTGAGCTGATACCGCTCG
CCGCAGCCGAACGACCGAGCGCAGCGAGTCAGTGAGCGAGGAAGCGGAAGAGCGCCCAATACGCAAACCGCCTCTCCC
CGCGCGTTGGCCGATTCATTAATGCAGCTGGCACGACAGGTTTCCCGACTGGAAAGCGGGCAGTGAGCGCAACGCAATT
AATGTGAGTTAGCTCACTCATTAGGCACCCCAGGCTTTACACTTTATGCTTCCGGCTCGTATGTTGTGTGGAATTGTGAGC
GGATAACAATTTCACACAGGAAACAGCTATGACCATGATTACGCCAAGCTTGTTGTAAAACGACGGCCAGT

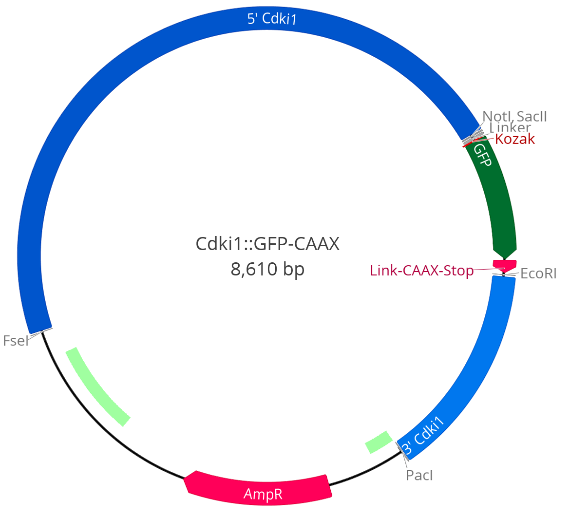

GGCCGGCCATT==**5’Cdki1**=TTACGCGCTCAAAATATAAATCTGTAGGATCTAAACTTCAAGCAAGAAGAATCTTCTTCTAAA
AAATATTTTTAGATTTTCACAACAAAGAATGTTTTCGCAGATTTTAGAAATATTAGGATTACTCCTCGAACGCTCTGCAGGCA
GTTTAACAATAACAAGAGGGTTTTGTTGTTAATCATGACATGCTGGCTTGGATTTATATAATTCAGTTTATAACATATAATAAC
TGTCCATCACAGTTTGAAAGAAATTAAAAGCGAAATGACACTCAATTGATCTTTTTCGACAATAAAAATAAAAAAGGATCAAA
CTTAAGATGAGGTGACCTTCAGACGAAATATAAACAAACAAACTCGGTTTTGCGTGGACGGGGCGCGGATGTGGTTTTTTA
TGGTTCAAACATTTTAAATACCTTCCCTAAAAAAATGTTAAGAAAACTTAAAAGAACTTCCAATGAAATTATGTGACCATAGA
CATCACGTTTAAACGAGCATGTAGTACTTTCATTTCCTCGCTCGCACAAAGTAATAATAGCACCACATTCCTTTCACTGTGG
AAGCTTATCACGTATCAACTCTATTTTTCTTCAACTATAAAATCTTTTTTGAGTATATTAATAAAGCGCTGTGTGTAAACTAGC
CTTTACTTAATTATTCAAAGATATGGTGATTAGGCCTTTAAACAATAATCTATTTATTTAAAAAAAACCTGGTAGACAGCTGTC
AAGTTATAAGCTATTAACCGTTATCAAGTCACAATAGATTTTTTTTAATCGGCACTTGTCACTTCGCATTTGAAATAGGTGTT
AGATTTTTTTACTATTGATTCCTGGGTTCCTAAATTAAATTAGAAGCGTATGAATAAAGCTGTAATCGGTATTTCTTTTTATAG
TTGACATCTGCTTACTGGTACAGTTTTGATTGAAACAGTCACTTTTTAAGGTTTAAAACCTTTTGGGGATACATTGGACTCCA
ATACGGCTGATGGTATACCAGATACATAACTAAAGTATTCAAAAAATTTTAAAGTGCCAGCTTGAATATTTTATGCCTTGATT
GTGATTTTTTTTTCGTTGTTTTAGACACTAGCAACAAAAAGGGGATAGAGGAGAGATCGGGGGAGAGAAATAATGCAAATC
TAGAGTTACTAGGAAACAATCCTGTCCTAACTGTTTCACTCTCATTTTATTATTGTGCAACTACATGTATGTTTTTAAATAACT
TCAACATCAACCATTTCTTTCAAACTCTAAAAAAAATCATTACCATCTGAATATATGTTTCCCTAATTTTCGGAAGAACTCAAT
TAGTAAAGACATATTTGGGACTGAAAATATGTCAAAAATAGGCATATTCAACGAGGTTAAATTTAGACGTTCTTATAAGGAAA
CACGTATCTTTGTTGTTTTCTTAGCTATGTAGGGTTTTTACACATTTTAAAACAAGTAATTCAACACTCTATGACTCCTCCTTC
TCCAAGATTATTATTTTTCAATATCATGATTATTATTTCTACACGCGAACAGATGTCAGTCAAGGAAGAAATCTTTTTATGATT
TGGTAATAATGTATATTTAATTAGCCATTTTAATAAACTTGAAAAAATTTAGAAACTTTGATAACAGCTGTCGCACATTATAAA
GGACCAATTAACTTATTTTCAAAAGTTTTAGTATTTCAGGGCTAGCTTTCTAATGTGAAGAGTGGTCGATGTGAAGAATGTC
AGGAAATTTGCGGCAATATTTAATAAATTTTATCTTCGTGAAAGATTAAACTGTTTTGAATGCATTACTTAGAAAATGTTCACC
TGCGAGTTTATATCTGGAATCTTTTTTAAAAAAAACCATTATTGTAAATTAGTTAGTAAATACATCAGGTTAGAACTAAGTACG
TCACGAGGAAGAATGGATCGCATGAGATGAAATCTGTTTCAAAATGACCGCACGATAACAAAATCTCATTCCTCATGAGAT
GTTTTTATTTTTTCGTGACTTGCATCGTCAGGTTGAAGTCATGAAATATAATGGTGATGTCTTATGCATCACGAAATGTTTTT
TTAAGATTTACGTCAACAATTACACGTTGTTCTTTCCCTATAAAAAAACACTCAAGAAACCTTGAAATGTTCTTAACTCTTCCC
ATTTAAAGCCTAACTATATTTCTAAATTGTTCTTAATGTTCCTGCTATTTTGTCATTTTTGAAGTTTTAAAGATATATTTAGTAA
TTTTCAGGTATTGACAAAGCCTTAAATGTTTTAAACATTTTGCCTTTTGACATCGAAGCCGGCAACGAAAAAACTTGTTTAGC
CGTCAAGTAAGCGGTAGCGACTTTGACATAGGCAACAAACTATGGGGACGAGGATGCGATCTATGTTGACCGATCGCCCG
ATTTGACTAATTTTTAACCTCGTTAAAGGGGTTGGACCAATTTTGTTTCTAGTGGAAACCCTCCTTAAGACACGCTTGTTTAT
CTTATGCTCTCTCCTCGTAAAAGTTGTTTCAAAGAGTGGTGATGAATTAATGCAATAAGGGTAAACCGAATTAGTACCGCTT
TATTGTGAAAATATCTATATGATGCATCATCTTTGAAGGTAAAAATTCTGATAGCATGTTCCGTCATTAGTTGAAAACCATGT
TTCATGCATGGCAACATTTGAGCTGTATATAATCGAATTGCTTGGTTGTACTTTCAGCAACATGACAGGAAGTACGCTTGAT
TAGTTTGATTAGCAGTGACTGACCACGAAATGTCAGTATGGTAAACCACATGGTTAGTTGTATAACGGTTTTTGTATTCACA
ACGTGTCGCGCAAAAAAATACGAAAGTTTTAATCTCTTCGTAAAAAATAGGTTTTTTTTAAAAAAAATTCAATCGGTAAAATG
TTCTTAAAAATAAACAATTAATATAGATCTAATAATAAATATACAGAAAAGTACAAAAAAGCTGCAAATTATTTTTTTTTAATAA
GAAGAAAATCAACCTCGTCTCCAGGACATTTTGCGAATAGCATTTGATTCTGGGTTAGGTTGGAAATAAGCGAAATAGGGA
AGTCTATGTTATGGGAAAAAGTGAAATTCTAGTGAGTAAAGATGGGGTACAAATATTATGTATTCTCAGGGTGACTTTTTAAT
TTGATGTGACAGAAAAGAAATTTGATATTATCCCTACGCCACTTATAATAAGTCTATCGAATGTTGTTCACAGCTTAAACTCT
TTGTTTTTACTTTGTTTTAAACTTGATCGTCGCGAGCCATTGAAAAATAAAAGATGAATAATTGATAACCTCCGCGATCAAGA
GACCAACCAACCACTGTACAGCAACATAAACCACGAGAAACTAAGTTGATATTTACAGCAATGACCTCTGGTTGTGGTCCC
AGTACTACTTTTGTCAACAACTTATCTAATTAATCTTTACCTATTATTTATTTATCTTAAGATTATCAAACAAATCTAGTTTTCA
AATATCTGAATTTTAAAACAAAAATAAAGTTTGTTTTTTAATGTGCTTCAAAGTATTTTTCAAAAAGTTCGCGCGGTAATTTTTT
TTTTCAAAGTTTACCTCTAGAGGTTTAGTGTCAGCTGTGTTCTTATTAAACTATAGCACAGCGGGAAATTTAAACAGAAACG
GCAAAAAATTCTAAAACTACTTAACACACACAGCTGGTAATTGTTGTTTTTCTTCTTCCCGCCAGTAGGCATAACGTTTAAAA
TACAATTAAAACACAGGAACCTATGGGGAATACCTTTCAAATAAAACAATAGAAACACAAAGTGTTATTAGCCAATCAAATCA
AAGTTTATTTGATCTTTTAGCCAATCATTTTTATTTATTATTTTATGAATGGCTTTTGTTATGTTTTGATCTGTTCATATGATCA
GACCAAAAGATTATGTTGAGTAGTTCTTGTTTGGCCACTGAGTCAGCGGTGCAAAAAGCTTTTAAAATTTAAAATTTAAGGA
GGGAATTTTGTTATAGTAATTTATAGTGATTTATAATAATACGAGTCGCGGCCGCCCGCGGTGCAGCCCCGGTAGAAAAAG

CCACC==**GFP^{CAAX}**=ATGAGTAAAGGAGAAGAACTTTTCACTGGAGTTGTCCCAATTCTTGTTGAATTAGATGGTGATGTTAAT
GGGCACAAATTTTCTGTCAGTGGAGAGGGTGAAGGTGATGCAACATACGGAAAACTTACCCTTAAATTTATTTGCACTACT
GGAAAACTACCTGTTCCATGGCCAACACTTGTCACTACTTTCTGTTATGGTGTTCAATGCTTTTCAAGATACCCAGATCATAT
GAAACGGCATGACTTTTTCAAGAGTGCCATGCCCGAAGGTTATGTACAGGAAAGAACTATATTTTTCAAAGATGACGGGAA
CTACAAGACACGTGCTGAAGTCAAGTTTGAAGGTGATACCCTTGTTAATAGAATCGAGTTAAAAGGTATTGATTTTAAAGAA
GATGGAAACATTCTTGGACACAAATTGGAATACAACTATAACTCACACAATGTATACATCATGGCAGACAAACAAAAGAATG
GAATCAAAGTTAACTTCAAAATTAGACACAACATTGAAGATGGAAGCGTTCAACTAGCAGACCATTATCAACAAAATACTCC
AATTGGCGATGGCCCTGTCCTTTTACCAGACAACCATTACCTGTCCACACAATCTGCCCTTTCGAAAGATCCCAACGAAAA
GAGAGACCACATGGTCCTTCTTGAGTTTGTAACAGCTGCTGGGATTACACATGGCATGGATGAACTATACAAAAGTGGACT
TAGATCAAAATTGAACCCCCCGGACGAATCGGGTCCTGGTTGCATGAGTTGCAAATGTGTATTATCATGAATGAGCTCGAA
TTCGAATTC==**3'Cdki1**=ATGTTCCTATTGGTTCGAAAACACGAAAGCATTTCTTCCAAGAAGATATAAATTTAAATATTTTCAT
TTTGAAAAGACTATTTTTTGCAAAGTTTTTTTTTGTTTGTATTTTTTTACATCATAAATTACCTACATGATGTAATCCCACCCTC
CCTTGTTTTAATATATACAAACATTATCCTTATTTACTTTGTCAAAAATTACATATTTTTTCAACAAACATATTATAGTGCGTGC
GGCCATTGATGGGCAACGTTATGTCAATATTTAATATCGTACGATATAATACATGTATTTTTTTGTCTGTAATATTATAAATTT
ATTTTTTATGTCAGACTTATTGTTCCTAAAGAAATTGTATATAAAACAAACCATTTAACGTATCATTTGTATATACGATCTTAAT
TTATGTGATAAGAATCCGGCATATGTCGGTTCATACCATATCTGTTGTTTTTGTTGCGTTCACGAAGTGTCTGAAAATTAAGT
GTCACTTGAATGACGGATATTAAAAAGGCGGGAATCATAGATTCGAGGGATAAGTGAAAACCAGTCTGTCTGATTATTTGC
CAAAGGAGGAGGAGTTGATTTATCTTAAGGATTTTATAATAAGCTTTGTTATACATTCTTAGCTGTTAACGTTGACATAAAAC
CCTGCTAAAGGTTTCATTTTTCAAATATATTTATTTGTCAAGAAAGAATTTTTTTTACCTGTGGTATGTTTTTATTAACTGAGA
CAAAAATATAACAACACCAATATAATCTTGGCTTAACCCTTTTTTTGAAGAATCCCAAATTATCAATATTTTTTAACAAATAGC
TTTAAATTTTAAACCGATTTCTGGCCACCTCCTAACTCAAGGCATTCTCGTCTTCAGAGCTCTTTGAGATTAAGACTTCGAGT
TTATCTCAAAGAGCTTTGGGGTCTAGAATGAACTCAAAGTAGATGAAAGTGAACACTGTCGGATGGGAATTTTGGTGTAAG
CCTCATCTTGATATCTTAAAGTTAAGTGATCACGATATATGGCTAATAGACGTTAAGTCAGTATTTCAGACTTAATGAGACGT
ACTTTGGAAGATTTATTGACAAACTTTTTAGAGGTGACGCGAAATATGGTGTAATAACATCAGTTATAAATATTTTAAAAATC
AACGATAACTTCAATCATATTTTCTTAAAATTTAAAGTAAATTTTAACATTAATTAAATTAACGTACGGGCCCTTTCGTCTCGC
GCGTTTCGGTGATGACGGTGAAAACCTCTGACACATGCAGCTCCCGGAGACGGTCACAGCTTGTCTGTAAGCGGATGCCG
GGAGCAGACAAGCCCGTCAGGGCGCGTCAGCGGGTGTTGGCGGGTGTCGGGGCTGGCTTAACTATGCGGCATCAGAGC
CGATTGTACTGAGAGTGCACCATATGCGGTGTGAAATACCGCACAGATGCGTAAGGAGAAAATACCGCATCAGGCGGCCT
TAAGGGCCTCGTGATACGCCTATTTTTATAGGTTAATGTCATGATAATAATGGTTTCTTAGACGTCAGGTGGCACTTTTCGG
GGAAATGTGCGCGGAACCCCTATTTGTTTATTTTTCTAAATACATTCAAATATGTATCCGCTCATGAGACAATAACCCTGATA
AATGCTTCAATAATATTGAAAAAGGAAGAGTATGAGTATTCAACATTTCCGTGTCGCCCTTATTCCCTTTTTTGCGGCATTTT
GCCTTCCTGTTTTTGCTCACCCAGAAACGCTGGTGAAAGTAAAAGATGCTGAAGATCAGTTGGGTGCACGAGTGGGTTACA
TCGAACTGGATCTCAACAGCGGTAAGATCCTTGAGAGTTTTCGCCCCGAAGAACGTTTTCCAATGATGAGCACTTTTAAAG
TTCTGCTATGTGGCGCGGTATTATCCCGTATTGACGCCGGGCAAGAGCAACTCGGTCGCCGCATACACTATTCTCAGAAT
GACTTGGTTGAGTACTCACCAGTCACAGAAAAGCATCTTACGGATGGCATGACAGTAAGAGAATTATGCAGTGCTGCCATA
ACCATGAGTGATAACACTGCGGCCAACTTACTTCTGACAACGATCGGAGGACCGAAGGAGCTAACCGCTTTTTTGCACAAC
ATGGGGGATCATGTAACTCGCCTTGATCGTTGGGAACCGGAGCTGAATGAAGCCATACCAAACGACGAGCGTGACACCAC
GATGCCTGTAGCAATGGCAACAACGTTGCGCAAACTATTAACTGGCGAACTACTTACTCTAGCTTCCCGGCAACAATTAAT
AGACTGGATGGAGGCGGATAAAGTTGCAGGACCACTTCTGCGCTCGGCCCTTCCGGCTGGCTGGTTTATTGCTGATAAAT
CTGGAGCCGGTGAGCGTGGGTCTCGCGGTATCATTGCAGCACTGGGGCCAGATGGTAAGCCCTCCCGTATCGTAGTTAT
CTACACGACGGGGAGTCAGGCAACTATGGATGAACGAAATAGACAGATCGCTGAGATAGGTGCCTCACTGATTAAGCATT
GGTAACTGTCAGACCAAGTTTACTCATATATACTTTAGATTGATTTAAAACTTCATTTTTAATTTAAAAGGATCTAGGTGAAGA
TCCTTTTTGATAATCTCATGACCAAAATCCCTTAACGTGAGTTTTCGTTCCACTGAGCGTCAGACCCCGTAGAAAAGATCAA
AGGATCTTCTTGAGATCCTTTTTTTCTGCGCGTAATCTGCTGCTTGCAAACAAAAAAACCACCGCTACCAGCGGTGGTTTGT
TTGCCGGATCAAGAGCTACCAACTCTTTTTCCGAAGGTAACTGGCTTCAGCAGAGCGCAGATACCAAATACTGTCCTTCTA
GTGTAGCCGTAGTTAGGCCACCACTTCAAGAACTCTGTAGCACCGCCTACATACCTCGCTCTGCTAATCCTGTTACCAGTG
GCTGCTGCCAGTGGCGATAAGTCGTGTCTTACCGGGTTGGACTCAAGACGATAGTTACCGGATAAGGCGCAGCGGTCGG
GCTGAACGGGGGGTTCGTGCACACAGCCCAGCTTGGAGCGAACGACCTACACCGAACTGAGATACCTACAGCGTGAGCT
ATGAGAAAGCGCCACGCTTCCCGAAGGGAGAAAGGCGGACAGGTATCCGGTAAGCGGCAGGGTCGGAACAGGAGAGCG
CACGAGGGAGCTTCCAGGGGGAAACGCCTGGTATCTTTATAGTCCTGTCGGGTTTCGCCACCTCTGACTTGAGCGTCGAT
TTTTGTGATGCTCGTCAGGGGGGCGGAGCCTATGGAAAAACGCCAGCAACGCGGCCTTTTTACGGTTCCTGGCCTTTTGC
TGGCCTTTTGCTCACATGTTCTTTCCTGCGTTATCCCCTGATTCTGTGGATAACCGTATTACCGCCTTTGAGTGAGCTGATA
CCGCTCGCCGCAGCCGAACGACCGAGCGCAGCGAGTCAGTGAGCGAGGAAGCGGAAGAGCGCCCAATACGCAAACCGC
CTCTCCCCGCGCGTTGGCCGATTCATTAATGCAGCTGGCACGACAGGTTTCCCGACTGGAAAGCGGGCAGTGAGCGCAA
CGCAATTAATGTGAGTTAGCTCACTCATTAGGCACCCCAGGCTTTACACTTTATGCTTCCGGCTCGTATGTTGTGTGGAATT
GTGAGCGGATAACAATTTCACACAGGAAACAGCTATGACCATGATTACGCCAAGCTTGTTGTAAAACGACGGCCAGTGGC
CGG

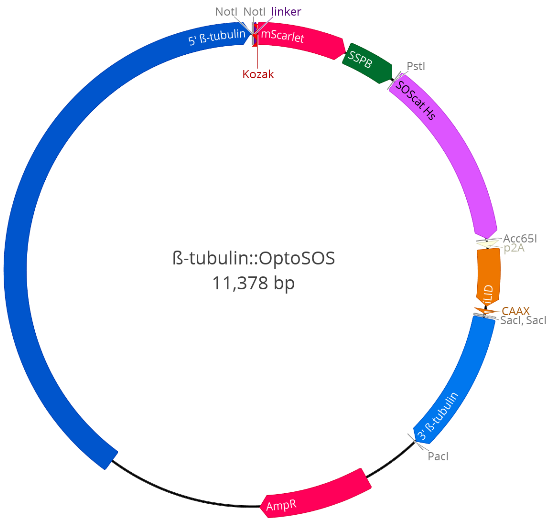

==5'ßtubulin=ACAACGATAAACAAACATAGCGTTATGCTGTTGTTTTTTTCAAAAAAAAATTTATACTAATCAAGTCTTTTTATC
ATGTTTTATAAATTCAGCGTTTTTATTTTTCCCTGATTCAAGCGATAAGGTCATCTGTTTGTTTTTCACACCATCATGTCTTCA
AAAGACGTACATAAGTTTTCGTGTCATGTTCACATGACAACAATTAAATAGATTTTTATACTGCTTAGGGTTTGTCAAAAGGA
GTGCATTTTTAACTCCAATAGGGTAGTAACAGCAAAGTGGATAAATCCTTTTCTTTGACAGTTGTAAGGCTAGTATCAAAGT
ATTGTTAAGCTTCAGGGTTATCTGACACGAAATATATTGTTCTCTATTACTCAGACGGTGTTTTCATTACCTTATGTTTTATAC
GGTAAAAAAAGTTTTAATCTTAAGCAATTATTTTAGCATCTCCAGATGCAATCTTTTGTTGTCTGCAAAAATCTAATTAGAATT
GTCATTACGTTTCCATGACAACTCTTATGAAATTACTGCTATCAATAAAAGTCATTAATAAAAGCTGTTTCATGGTCACTGAG
GAGCTTTGTAAAAACTTTACCAGTAGAGGGGCGTAACATTTTCAGATAATAGCCATCTAGAACCCAGTTTTCTGATGCTTAA
AAACCTGGGTACAGAAAGCTAGTTTATTCTAAACAGCTACGAAACACGTTTTTACCCAGATAATTTCTTTGTAAATTTCCTGC
CCGTTTTCTCTTTAAAAGAAAGACTACTTTTTTAATTTAGTGACTTACACGAAATTCGAATAACGCGACAGAAATTGTTAATA
CTGTTAATGTTACACGTAGAAAACCTGTAGAGTTCTGCCATTTTGAATGTGTTTATTTGTTTTAGATGACGTCACAAGAATTT
CATGAAGATTTGGATATGTTGCTCAACATGGAAATGAAGTTATTACTGCTCGATTTAGAAGATGTTCGGATACCGGAAAATC
CTCCCGCAATACCTCGTGAGCCGGAAAATTATAATTTCTACTACAAAGATGATTAGAAACGATAACTGTCTACTTTGTCCTT
CCAGAAACTTTATCGTTTTTGCTCAATCCAGCTATTTGAACGTTTTGAATAATCATCGAGTGAACGAATATGGCGAAAGTTTT
CTTTAAGTAATTACAAAAATTTAAATTACGAGGGACCAGAACTTAGTCACGTGACTGGTGACAACGCATACTCTTTTATAAAA
AAATTTTTCTGCCTCAAAGCAATAATTTAATTATAAAAATTTCAAATTCTTTTTTGCTTGTTTCTGCTCAGTTTTTTACTGAAAA
GGATTTATTTGCAGCAATTTTAATTTAATCGCCGTTTTTTTCAGTTTGGGCTTTTTTAAAACGATCCATGTAGGTTGAATATTC
TTATAGCAATCGATTCAGCTTTAAGTGGTTTTGAATATTTTACCATATAAAAACAAAAAAGTTTGCGGCTGCAACAGACCGAC
ACTTGGTCTCTGTTATCTCAAATTACCTGGAAGGTTAACTGTTTTGCGATGTTGTTTTTCTTCACCAGTGATGGCTTGAGCA
GAGGAAACTCACGCAGCGTCCTCAAAAGACATTTCAATTTCAATGCTTTTTTGTCGCAACTATTGTCTATATTGTGATAAAGA
AAGATACTTTTTTTTTTAAAGTCTGAATATAAATTTTTGTAATATTTTTTTTCTAAAAAGATGACAGCAAAATTGTGTAAAAATA
AGAATTGTATAAACCTATTGTGTTTTAGTGCTTTGAAATTTATAAAATATTTTTTGTTGACAGTTGCAACTTTGTACTTGTATAT
ACACATCTCAAAATACATAATTGTAAAAATTCGCTGTGAACTATTTTTATCTGCTCCCTCTGGTCCACATATTGCATATCTAAT
ATATATTCTCTCGTTTTGAGAGATCATTGTTAGATCTTATTATTTCCGCGATAACATTTTAATAAAATATATTTTATCGGTTGAT
CCTACTGACAGATGCTTCTTATAACAGCCGTCCAATATTCTTCATTAAAATTATATTGCAGAACCCACCTTTAAAACACTCCA
GGGTGGAATATATTTATTTACTAAAATAAAATTATGGCTTATCCTACATTATTTAGTAATGAGAGTTTGAGCACGGATCACAT
TTACTTGTCGTTTTTGCATAAATATATTTGGCACCTCTCATCACTGGGTGGAAAAATTTCATTATGGTTAGCTTTTCGGAATT
TTTTAAGCGGCAGATTGTGTTTGTGGTAGGATGTTCAAACATCTCTTGTTGTTGAAACTACCAAGCTAGAATTGAAAATAATA
AATCATCGAAGATTCCTACGGATTTCCGAACTACATTTTTGTTTAAGGAGTTTTGTAAATTCCACATGTGGACTTTGAATGTT
TTTTACATTCTGGAAAACTATTTTTCTGTGAGAAAAACAAAATTTGAATTAGTTATGCTTTTAAAAATATTCCTTTTGACATTAA
AACGTGTTGTCTAAAGTAAATTGAAAAGATCTCGCCTCATAAATAATTTCTATTCAACCCACAAATTTTAATTCAGTACGTATT
CGCTTACTTGTAAACGTAATTGCCACATACATAATTTACTGTTTATTATTCTTGATGATGAATATAAACTTCGAAACGTGCGTT
GAATTTTTATTATGAATCGAGAAAAATCAAAATAAATTAAACCGCTCGTGAAAACCACTTCCACCGCTGTTTAACATTGAGGT
GAAAACCTATTAGGCACAACCCTCTAAGCAAAGACCGAAAAATATCCTAGACTCTAGTTTAAGCTTTTAACGCCGTACCAGA
AAACGTTTAAAGTACAGCGATTTCTTTGTTCAACTACATATTAAAGCCAACCTTGTCCCCAGGGCCACTTGTCTTTTACTCTA
CGCTAAAGAAAAAGGCGAAACAGTTCCTGGGCACGAAGTTGATATGAATATGTTCCCTGGTAACTTGCAAATAAACGTACG
ATCGTGATTCTCAGCTGATGATTGAATGATAACAATCTGTTTTTATGGATGTTCTCAGGAGGAAATTATAGTCGATTAAAAAA
AATGTCCGTTGTGTTGCAATATGTGTTTTTAATTAGCAGGTTTTTAAGCCAATTATATATAGGACTGTTAATTGGTTTGACGA
GTAAACAACTACCTTCTCGCAGCAAAATGCAAAGTAGCTGCACAACCTAAGAAACTAAGAAAACTTGGCTGAGATTTTTTTT
CTAAAATTTAAAAACAAATTCCGGAACAAAATAATGTGAGTCTCGAAATACTAAAAACTATTTTTCTCGGAACATTAACCGTG
CTATCTCTTCCAATCCTATGAAGTGTTCGTCATCTTCTGTAAAATTGACCCAACCTTGTCCCTAGGCTAGTTAGGTTTGTTTA
TATTTGGACGACCGTCCAAAACACCGAAGAAGCCCTGGGGACGAGGTTGAAACTGACTATCCGTTTTCCCTGTTTTTAGAA
GAACCCAAATATGAACATTCGAGACTGAAAATGTGATTTTAAGCATGATAAAAAAGAATTAACATGTCGAGAGCTACTATTT
GCTTCTCCTCCAGACCGCTGAAGAAATATTTTTCCGCTTTGGCACGACTTTGTTTGTGGTGAGCAAACACGAAAGCAAATG
CATATTGGATGAATTTAGATTTATGGATGCTGGTTGAAAAACAGCGTGAATTATTCTGCAAACGTAATGTAACTTCGAATTAT
TTATTAACAAAACAATGTATAGTCGCAGCTAGATTTTCAAACTCTGTCTTTTTCCACGATCCTATCAAATACTCGCAAGAAAT
GAAAAAAACTCAGTTTGTAGTCGAGCCGGCTACAGCTAGTTAGCTATTTGTACATAGTTTTTAATTTGTCATTTTTATTAGTG
CCACACCCCAAAACCTCTTCTAACAAAAATAAACCCCATGTTACTTAAAAAAGAGAGAATTAGAGGGCGAAAATTTCCTGCA
TCGCTAAATCATAAGAAACATGTATTATTTATATCCAGTTGTCAACTCGGTCCACAGGGTAAACATTGCTCGGCTGTCATAG
TGATAAATCTACTTTGCATTATGTTGTCCGAACAACATAACAAAAAACGCGCCAATCTTTCTTCACTAGCCAATTAAAATAAA

CATCCAAAGCACTTTAAAAGTTGATTGGCTTATAAAAGAACGCACTACAATGAACGCCTCAGAAAACAGACGGGGGTTTTAT
TAAGCATTTAGAGTTTAAGAATTTCACCATGTTGCCAATATTTCAGATAATTTAAAGTTTTTTTATATAAAAAAATATTTTTTCT
TATATTTCATGCAGAATTTATAATTGATGTTGCTCTAGTATAGGATCTATTTGGTAATGATTTTCAAATCATTACCCCCCGGTT
ATACTTTTTCTATAGCAACAAGAGGAACCGATTTTGCACAACCATCACAAATCTTTCATTGCAAGTCGCAGGCGGCTCAGAG
AAGCTCGTAAAAAAAAACTCTTACAAACTAGGTGTTGTAGCACGAACTATATAATTTCAATGCGGCGGCCGCGCGGCCGCGG
TGGAGGTGGATCTGAAAAAGCCACC==**mScarlet**=ATGGTATCTAAAGGTGAAGCAGTTATAAAAGAGTTTATGAGATTTAAA
GTTCATATGGAAGGTTCCATGAATGGACATGAATTTGAAATTGAAGGAGAAGGTGAAGGTAGACCATACGAAGGAACACAA
ACAGCTAAGTTAAAAGTTACAAAAGGAGGTCCACTGCCATTCTCGTGGGATATTTTATCTCCTCAATTTATGTATGGTTCCA
GAGCTTTCATCAAGCACCCAGCTGATATACCGGACTATTATAAACAAAGTTTTCCGGAGGGATTCAAATGGGAAAGAGTTAT
GAACTTTGAAGACGGAGGAGCTGTTACAGTAACCCAGGATACCAGTCTGGAAGATGGTACTTTGATTTACAAAGTTAAATTA
CGAGGAACAAATTTTCCGCCTGATGGCCCTGTAATGCAGAAGAAGACAATGGGTTGGGAGGCATCGACTGAGCGTTTATA
CCCCGAAGACGGCGTCTTGAAGGGAGATATAAAAATGGCTTTACGATTAAAGGACGGTGGCCGTTATCTAGCTGATTTTAA
AACGACATATAAGGCAAAGAAACCCGTTCAAATGCCTGGTGCTTATAATGTTGATCGGAAATTAGATATCACATCACACAAC
GAAGATTATACAGTTGTCGAACAATATGAGCGAAGTGAAGGCAGGCACAGTACTGGAGGAATGGATGAACTATACAAG==**S**
SPB=AGCGGGCTTCGCTCAAGAGCACAAGCCTCTAACGAGTTCGGTATAGATCTAAGTGGACTCACACTCCAAGAGTTTTC
GTCCCCAAAACGTCCTAAACTTCTTCGAGAATATTATGACTGGTTAGTGGACAACTCATTTACGCCGTACTTGGTCGTAGAT
GCGACCTACCTGGGTGTCAATGTACCAGTTGAATATGTCAAAGATGGACAAATAGTTCTGAATTTGAGTGCTTCCGCAACA
GGTAATTTGCAGTTAACTAACGATTTTATTCAATTTAACGCAAGATTCAAGGGAGTTTCTAGGGAATTATATATCCCAATGGG
AGCAGCCTTAGCAATTTACGCTCGAGAAAACGGCGATGGCGTTATGTTTGAACCCGAAGAAATCTACGATGAATTAAATATT
GGTTCAGCTGGTGGAGAATTTCTGCAGATG==**SOScat**=CTACACACTCCTGATCCCTCTCTGTACAAATTTGCCGAGGAGG
ATTCGGATGAGAATATAAAATTTGAAGATTCTGGAGAGGATGACACTGGTATTCCTGTTTTAAAAGGTGGCACATTGTACAA
ACTAGTTGAGAGGCTGACATATCACAAATATGCTGATCCAACATTCATTCGTGTGTTTTTAACAACATATCGCTCGTTCAGTA
ATCCTGTTGAACTTCTCGATTTGTTGATAGAACGTTATGATATTCCCGAACCAACTACGCTAGCCAATGGAAGTGACAAACC
TGTCGTTACAAGGGAGGAGTTGAAGCGATTTCGTAAGGAATATTCACATCCAATACAGCTTAGGGTACTGAATGTGTTACG
TCATTGGGTGGACCAACATTTTTACGATTTCCAAAGAGAAGAGACTCTATTGAGTACATTACAAAAGTTTTTGTCCCGAGTA
GTACGCAGTAAAACGGCGCGAAAATGGGTCGAAACCATCGAGAAGATTGTGAACAGAAGATTGGAAACAGCATCAGTGGG
ACCGGAAGTCTACACATTCAGCACTCCCGCACCTGCTTTCGAATGGCATCTTACAAATAATATCGAAAAATTCTCTTTACTC
ACTCTTCATCCGTTGGAGATTGGTCGTCAATTAACCATCATGCAATCCGAAATATTCAGATCTATCAAACCATCTGAATTAGT
TGGAACCGTGTGGGTGAAGAAGGACAAAGAAAAGCTCTCTCCCAATGCGTTAAAAATGATTCATCTCTCTACTTTGTTAACC
TTTTGGTACGAACTCAGCATATGCGAAACGCACAACTTTGAAGAACGAGTTGCAGTTTATACTAGAATTATTGATATATTAAT
GGTGTTTATCGAATTGAATAATTTTAATGGTATGATGGAAATACTTGGCGCACTCAATTCGGCTCCAGTGCATAGACTTCGC
TTCACAACAGGAGAACTTTCACCGAAGAGAATGCAAGCACTGCAATACGTGAAAGAATTAACAGATGACGGACACAACGTG
AAGTACACGGAGAAACTACGTTCTATAAACCCTCCTTGCGTACCGTTCTTAGGTGTGTACTTATCCAATATTTTAAAAGCGG
AAGAAGGTAATCCTGACTTTTTACCAAATTGTCCAGACGGCATCATCAACTTCAGTAAAAGGCGTATGGTAGCTGATATCAC
CGGAGAAATACAAAAATATCAAAACATGCCATACAACCTTCAGATTCAACCACAAATTAAAAAGTATATTGAGGAATTAGAC
CCCTATGCTGGTCGAACAGATCAAGAACTTAAAGATCACTTGTACGAGTCATCGCTTATGATTGAACCAAGACAAGCAAAA
CAACCAACGAAATTTGTAAGAAAATCAGACATACATCTTAAATCACCCGGCATAAAAGCTGGATCCAGAACGCAATCTATGG
GTGGTACCGGAAGCGGA==**P2A**=GCTACAAATTTTTCATTATTAAAACAAGCTGGTGATGTTGAAGAAAATCCAGGTCCA==**i**
LID^{CAAX}=GAATTTTTGGCTACGACATTGGAGCGGATAGAAAAAAACTTCGTTATAACCGACCCAAGACTTCCAGACAATCCTA
TTATCTTTGCTTCTGACTCGTTTTTACAATTGACCGAATACTCCCGCGAGGAAATCTTAGGCAGAAACTGTCGTTTTTTACAA
GGTCCGGAAACCGATCGTGCAACTGTAAGGAAAATCAGGGATGCGATAGATAACCAGACAGAAGTAACAGTGCAACTGAT
AAACTATACTAAGTCAGGTAAGAAATTCTGGAATGTTTTTCACCTCCAGCCTATGCGAGATTACAAAGGAGATGTCCAGTAT
TTTATTGGGGTGCAACTTGATGGAACAGAGCGATTACATGGTGCCGCAGAACGAGAAGCAGTTTGCTTAATAAAAAAAACT
GCATTTCAAATTGCTGAAGCCGCTAATGATGAAAACTATTTTGGTTCGGGATCAGGAAGTAAGAAGAAGAAAAAAAAATCAA
AAACAAAATGTGTTATTATGTAACCCGAGCTCTAACCCGAGCTCTC==**3’ßtubulin**=ACACTTTTCGTTTTAATTTATAATTTTA
AGTTTTGCTTGTATATCATGAATATAGGGAAGGCACAGAAGTAACGTAAATGATAGTATTATTCTTTATTCCAATACTTAATA
AAGCACAAGGCCCAAAATTTGCTATGGGAAACATTTACGCGCTTAAAACATAAAAATCAAAAGCCTCCGTTTCCAATTTTTT
GCGTTTTTTGAAATGTCATTTTCCTGTACTTGAGAAACCGAAAGATACTATATAAAAAAATGGGTTCAAACCGGTCGCCAGT
GGAAATCCTATCAACGGAAATTATACNNATTCATTGCTTCAACTAATAAGTTCGTATGTCGAATAATAAAATGATTTCAGACC
CAAGTTACGATTAACGGTTCTTGAATTATCCTATTGTCGCTAGAAATTGGTGCAACGACAAACCTCTAATTCGATGGCTCGA
GAATTCGAGTTATAGCACCGATTTCGCTTCACACATACTGACGGGTTTTGATGTTGCACTGTGAAATGCAAAGCTCGTCCC
ATCACCTTTAACCCTGGCTGTATTAGGGGCAGCGCTGTTGAGAAGAAAAAGTCCTGGGGACGAGGTTGAAGTCTTGTCGA
GATAACTTAGTTCATACTTTGAACTGGGTTGTACTACAGACACATCTCTTTGTGGACCTCTGTCCGAACAATTGATTCATATG
TTTAAAACCTACTGAATACCATTTTCTTACTATTTAAACTTCATTATTTTTGGGAAAGGCCATTGCTTAGATGCAACCTCGTCC
ATAGGGTTTTTGCCTTCTTGACATCAAGTAAGCGCTAGCGGCTCCTGGGGACGAGGATGGCTTAAATGGGGATTCATCCAA
CGGCCCACACACAGTGTACCATCTCCCCATTTGCCCTCACATGGTTAGGGGTAACCCGGGGCTATGGTAACATTCTCTCG
CCCATATTGTGAATAGCTGTCAAGGGAAACCGAACCCCGGTCTGCCGCACAAAGTGCGCGAGTTATAACCAAAGCTACGG
CGCCAGTTTTAAGGGCAATGTCCGTTTATGCCGAATGTGACGCCTCGGTTGCCCACGATGGCTCCTATGGCTCACTTAATT
AACGTACGGGCCCTTTCGTCTCGCGCGTTTCGGTGATGACGGTGAAAACCTCTGACACATGCAGCTCCCGGAGACGGTCA
CAGCTTGTCTGTAAGCGGATGCCGGGAGCAGACAAGCCCGTCAGGGCGCGTCAGCGGGTGTTGGCGGGTGTCGGGGCT
GGCTTAACTATGCGGCATCAGAGCAGATTGTACTGAGAGTGCACCATATGCGGTGTGAAATACCGCACAGATGCGTAAGG
AGAAAATACCGCATCAGGCGGCCTTAAGGGCCTCGTGATACGCCTATTTTTATAGGTTAATGTCATGATAATAATGGTTTCT
TAGACGTCAGGTGGCACTTTTCGGGGAAATGTGCGCGGAACCCCTATTTGTTTATTTTTCTAAATACATTCAAATATGTATC
CGCTCATGAGACAATAACCCTGATAAATGCTTCAATAATATTGAAAAAGGAAGAGTATGAGTATTCAACATTTCCGTGTCGC
CCTTATTCCCTTTTTTGCGGCATTTTGCCTTCCTGTTTTTGCTCACCCAGAAACGCTGGTGAAAGTAAAAGATGCTGAAGAT
CAGTTGGGTGCACGAGTGGGTTACATCGAACTGGATCTCAACAGCGGTAAGATCCTTGAGAGTTTTCGCCCCGAAGAACG
TTTTCCAATGATGAGCACTTTTAAAGTTCTGCTATGTGGCGCGGTATTATCCCGTATTGACGCCGGGCAAGAGCAACTCGG
TCGCCGCATACACTATTCTCAGAATGACTTGGTTGAGTACTCACCAGTCACAGAAAAGCATCTTACGGATGGCATGACAGT
AAGAGAATTATGCAGTGCTGCCATAACCATGAGTGATAACACTGCGGCCAACTTACTTCTGACAACGATCGGAGGACCGAA
GGAGCTAACCGCTTTTTTGCACAACATGGGGGATCATGTAACTCGCCTTGATCGTTGGGAACCGGAGCTGAATGAAGCCA
TACCAAACGACGAGCGTGACACCACGATGCCTGTAGCAATGGCAACAACGTTGCGCAAACTATTAACTGGCGAACTACTTA
CTCTAGCTTCCCGGCAACAATTAATAGACTGGATGGAGGCGGATAAAGTTGCAGGACCACTTCTGCGCTCGGCCCTTCCG

GCTGGCTGGTTTATTGCTGATAAATCTGGAGCCGGTGAGCGTGGGTCTCGCGGTATCATTGCAGCACTGGGGCCAGATG
GTAAGCCCTCCCGTATCGTAGTTATCTACACGACGGGGAGTCAGGCAACTATGGATGAACGAAATAGACAGATCGCTGAG
ATAGGTGCCTCACTGATTAAGCATTGGTAACTGTCAGACCAAGTTTACTCATATATACTTTAGATTGATTTAAAACTTCATTTT
TAATTTAAAAGGATCTAGGTGAAGATCCTTTTTGATAATCTCATGACCAAAATCCCTTAACGTGAGTTTTCGTTCCACTGAGC
GTCAGACCCCGTAGAAAAGATCAAAGGATCTTCTTGAGATCCTTTTTTTCTGCGCGTAATCTGCTGCTTGCAAACAAAAAAA
CCACCGCTACCAGCGGTGGTTTGTTTGCCGGATCAAGAGCTACCAACTCTTTTTCCGAAGGTAACTGGCTTCAGCAGAGC
GCAGATACCAAATACTGTCCTTCTAGTGTAGCCGTAGTTAGGCCACCACTTCAAGAACTCTGTAGCACCGCCTACATACCT
CGCTCTGCTAATCCTGTTACCAGTGGCTGCTGCCAGTGGCGATAAGTCGTGTCTTACCGGGTTGGACTCAAGACGATAGT
TACCGGATAAGGCGCAGCGGTCGGGCTGAACGGGGGGTTCGTGCACACAGCCCAGCTTGGAGCGAACGACCTACACCG
AACTGAGATACCTACAGCGTGAGCTATGAGAAAGCGCCACGCTTCCCGAAGGGAGAAAGGCGGACAGGTATCCGGTAAG
CGGCAGGGTCGGAACAGGAGAGCGCACGAGGGAGCTTCCAGGGGGAAACGCCTGGTATCTTTATAGTCCTGTCGGGTTT
CGCCACCTCTGACTTGAGCGTCGATTTTTGTGATGCTCGTCAGGGGGGCGGAGCCTATGGAAAAACGCCAGCAACGCGG
CCTTTTTACGGTTCCTGGCCTTTTGCTGGCCTTTTGCTCACATGTTCTTTCCTGCGTTATCCCCTGATTCTGTGGATAACCG
TATTACCGCCTTTGAGTGAGCTGATACCGCTCGCCGCAGCCGAACGACCGAGCGCAGCGAGTCAGTGAGCGAGGAAGCG
GAAGAGCGCCCAATACGCAAACCGCCTCTCCCCGCGCGTTGGCCGATTCATTAATGCAGCTGGCACGACAGGTTTCCCGA
CTGGAAAGCGGGCAGTGAGCGCAACGCAATTAATGTGAGTTAGCTCACTCATTAGGCACCCCAGGCTTTACACTTTATGCT
TCCGGCTCGTATGTTGTGTGGAATTGTGAGCGGATAACAATTTCACACAGGAAACAGCTATGACCATGATTACGCCAAGCT
TGTTGTAAAACGACG
